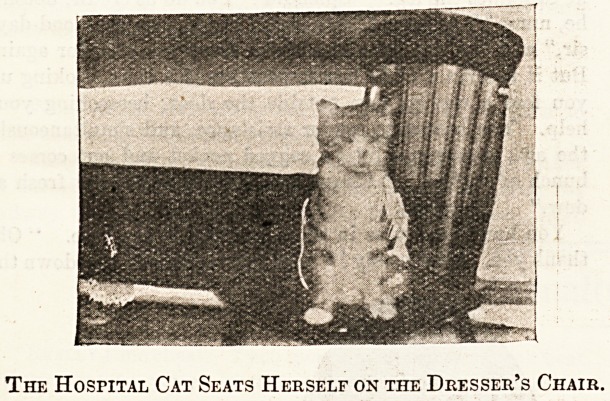# The Hospital. Nursing Section

**Published:** 1905-10-14

**Authors:** 


### The Hospital.

### Nursing Section.

Contributions for this Section of "THE HOSPITAL" should be addressed to the EDITOR, "THE HOSPITAL" NURSING SECTION, 28 & 29 Southampton Street, Strand, London, W.C.

No. 984.—VOL. XXXIX. SATURDAY, OCTOBER 14, 1905.

### Notes on News from the Nursing World.

### ISOLATION HOSPITAL BOARDS AND UNTRAINED NURSES.

IN sending us the remarkable article on "Nursing in Small Isolation Hospital," the author mentions that it would never have been written except for the comments on the nursing in isolation hospitals, and the vagaries of Isolation Hospital Boards, which have appeared in our columns from time to time, "proving that the editor is interested in the matter and aware of the need for reform." The statements of our contributor, following so quickly upon the heels of the account of a large isolation hospital without a trained nurse, certainly give point to the plea for searching reform in the present system of administration. The incidents which she is able to recite from personal knowledge should suffice to remove any lingering incredulity that may still exist in the minds of the sanguine or unsuspicious. But the pith of her contribution is contained in the declaration accompanying her account of the work of "nursing" in isolation hospitals that "the medical officer could not obtain the services of trained nurses, as the Joint Hospital Board would not pay them." This is putting the saddle upon the right horse. The time has come when public opinion must be brought to bear upon isolation hospital authorities in order to compel them to pay for the proper nursing of the sick under their care.

### THE ADA LEWIS NURSES.

WE are able to announce that Mrs. Lewis-Hill, more widely known in philanthropic circles as Mrs. Ada Lewis, who is founding an institute for the supply of daily visiting nurses for the poor of the middle classes, is quite prepared, if the initial effort clearly meets a real want to materially extend the movement. The opening of the Nurses' Institute at 62 Oxford Terrace, Hyde Park, on Thursday, October 26, may, therefore, in time to come be looked back upon as the beginning of a very important and beneficent work. As will readily be imagined, the scheme has not been hastily adopted. It matured a year ago, and the fullest possible consideration has been given to all the details. There is a strong committee, but as the means are provided exclusively by Mrs. Lewis-Hill, and her desire is only to benefit a class which is not systematically assisted by charities, no names will be published. While the primary object is to enable the sick poor of the middle classes to enjoy the advantages of trained nursing, it is hoped that the establishment of Ada Lewis Nurses' Institutes will tend to relieve the congestion of the hospitals for the benefit of the general practitioner. The nurses will be employed only at the instance of the medical men consulted by the patients. They will be experienced in district work, will, of course, reside in the institute, and will not take any meals in the homes of the patients. The boon which the organisation is likely to prove may be judged by the fact that the maximum fee of ten shillings per week covers attendance at an operation, which includes preparing the patient and the room, assisting the surgeons, and clearing away afterwards. The fee is, therefore, almost nominal, and, as no donations from any source will be accepted, the cost to the generous originator will, in any event, be substantial. She will doubtless feel amply rewarded if her meritorious attempt to help those, whose poverty is all the more grievous to bear because it is not paraded, is sufficiently appreciated to justify the inception of more institutes in different parts of the metropolis.

### GUY'S NURSES AT KANDY.

THE matron of Guy's Hospital has received from the hon. secretary of the Colonial Nursing Association a letter, from which the following is an extract: "The principal medical officer writes: 'The Kandy Sisters, led by Miss Fricker, are a great success, and this is due to Miss Fricker. We could not have obtained a better matron in any respect. The difference she has made in the nursing at Kandy is marvellous.' Sir Allan Perry is trying to get money for a fourth nurse at Kandy, and is particularly anxious that, if he succeeds, she should be trained at Guy's." Miss Fricker, who was the senior sister in the Preliminary Training School at Guy's, only left for Kandy in March last, taking out with her Nurse Wilkinson, while Nurse Corder, who had been working at Colombo, joined them on their arrival. That in such a short time the matron and her staff should have effected such a marked change for the better is a signal proof of the excellence of the training at Guy's.

### MECHANICAL WARD WORK AND INDIVIDUAL ATTENTION.

THE matron who is author of the article headed "Minor Nursing Amenities," which appears in another page, was impelled to write it after a month's experience as a patient in a London hospital. This experience made her feel very strongly that too much importance is often attached to mechanical ward work in our general hospitals, and that too little individual attention is given to the patient. She supports her belief by the narration of several incidents of which she was an eye-witness, although she does not include the fact that in the hospital in question patients are washed at four o'clock in the morning, if they happen to be awake, as an argument in support of her conclusions. No doubt it is true that many regulations are carried out because authorities do not realise that they may be a hardship to the patients. But as our contributor hints, even to the bustling nurse who had "no time" to listen to the patient, the comfort of those under her charge should not be such a very secondary consideration. The hospitals, after all, exist for their benefit, and not in order to provide nurses with useful training. Even, however, in the interests of the training of nurses, the comfort of the patient should not be overlooked.

### THE SUSPENSION OF PRIVATE NURSING AT SWANSEA.

A curious decision has been arrived at by the Swansea Hospital Board. They have determined to suspend the private nursing department for a time. This is, of course, a compromise between the views of the members who are in favour of the discontinuance of the department, and those who wish for it to be maintained. The House Committee, who advise the former, affirm that all the available accommodation in the hospital is required for the hospital nursing staff, and we certainly think that this is an irresistible plea against the heresies of the private staff in the building. We do not, however, see why the private staff should not be housed elsewhere. In other cases this course is pursued without any unsatisfactory results. If it be the deliberate opinion of the authorities at Swansea that the continuance of the private staff is in any way detrimental to the interests of the Hospital, then it should be done away with instead of suspended. But in the interests of the Swansea people it seems very desirable that private nurses controlled by the hospital authorities should be available.

### A CHARGE NURSE'S ALLEGATIONS AGAINST A MEDICAL OFFICER.

The official report following an inquiry instituted by the Guardians of the Stoke-upon-Trent Union into complaints made by Miss Birch, a charge nurse in the Spittals Workhouse Hospital, against Dr. John M. Longford, resident medical officer, has been forwarded to us. The charges against the medical officer were that he had punished the sister for alleged extravagance in safety-pins by sending her to a block where there were none but helpless cases, and it was insinuated that he desired to persecute her by hindering her from obtaining a post at Leicester. The superintendent nurse, the inmates of the workhouse, and other witnesses, supported the medical officer in his absolute denial of the existence of any foundation for either of these charges. It was affirmed that the only reason why the charge nurse was removed from one block to another was to give her lighter work; and that, instead of trying to prevent her from securing an appointment elsewhere, the medical officer had written her a helpful testimonial. The Committee, acting as a Committee of the whole Board, signified their opinion that the grounds upon which Nurse Birch based her complaints were "wholly inadequate and insufficient to sustain the charges she made," and proceeded to express their "utmost confidence in Dr. Longford, and their entire satisfaction with the manner in which he discharges his duties." The only possible explanation of the conduct of the nurse, who was not trained at Spittals Workhouse Hospital, and has only been there two years and a half, is that she appears to have been for a time in indifferent health. We are sorry that in any circumstance she should have allowed herself to make such odious accusations without the shadow of an excuse; but we are glad that the medical officer has been so completely exonerated.

### THE POSITION AT CARDIFF WORKHOUSE AND INFIRMARY.

With regard to our note last week headed "One Male Nurse for a Hundred Patients," the Clerk to the Cardiff Guardians assures us that we are misinformed, and that the state of affairs to which we referred prevailed in a block of the workhouse, not in a portion of the infirmary. It was in respect to the former that an inspector of the Local Government reported that there were a hundred male sick and only one male nurse to look after them. The Clerk to the Guardians states that the hundred patients in question are not sick in the ordinary sense of the word; they are "chronic and venereal cases, who need some attention," and "have for many years been under the care of two male nurses—one by day and one by night—who find among the patients themselves plenty of pauper assistance." The suggestion of the inspector, that another nurse is desirable, he intimates, is receiving the careful consideration of the Board, "but they do not feel that there is 'urgent need' to 'make haste' to alter an arrangement that has lasted so long without previous complaint or comment." The increase in the staff of probationers to which we alluded was, as we mentioned, recommended by the medical officer, and it has now been sanctioned by the Board, who have, however, for some time past contemplated the employment of additional probationers. But the additional probationers will work in the wards of the infirmary, under the superintendent nurse, with five charge nurses and eight probationers. This, for 188 patients, is satisfactory provision, but no provision for the hundred patients in the workhouse will be satisfactory, under which assistance admittedly needed is rendered by paupers.

### QUEEN ALEXANDRA'S MILITARY NURSING SERVICE.

We are officially informed of the following appointments and changes in Queen Alexandra's Imperial Military Nursing Service:. Miss K. A. Allsop, staff nurse, has been transferred from Aldershot to Princess Louise Hospital, Alton; Miss F. A. Dawson, staff nurse, from Alton to Cambridge Hospital, Aldershot; Miss E. Close, staff nurse, has been posted to the Royal Victoria Hospital, Netley; and Miss C. M. Williams, staff nurse, to the Royal Victoria Hospital, Netley. Miss A. L. Cox, matron, from Shorncliffe; Miss E. M. Denne, sister, from Woolwich; Miss E. L. McAllister, staff nurse, from Netley; and Miss

A. A. Steer, staff nurse, from Millbank, have been transferred to s.s. *Plassey* for Indian troopship duty. The following are held in readiness for service abroad: Miss L. E. Mackay, Miss E. S. Mason, and Miss W. Walker (sisters); Miss G. M. Smith, Miss E. M. Perkins, and Miss M. E. Wilkin (staff nurses).

### GIFTS AT GRIMSBY HOSPITAL.

It is satisfactory to learn that the bedrooms which were added to the Nurses' Home at Grimsby Hospital, in place of the cubicles which were at first proposed, are very much appreciated by the nursing staff. Both in their interest, and in that of the hospital itself, we recommended the course which the chairman and his colleagues so readily and graciously adopted. Our Commissioner, whose interview with the matron of Grimsby Hospital appears in another page, mentions that the whole of the contents of the nuises' sitting-room, from the carpet to the pictures, were given by individual donors. This tends to indicate the popularity of the institution in the town, and we are sure that the same result might often be achieved elsewhere. People who will not contribute a lump sum to a general fund will often give a particular article for a particular purpose. We believe that nurses' homes throughout the country, wherever they are wanted, might be more or less furnished throughout by appeals for individual gifts.

### DEATH OF A ROYAL RED CROSS HEROINE.

The death from dysentery on October 3 of Deaconess Jessie Ransome, of the Church of England Mission at Peking, has caused the utmost regret to a large circle of friends and admirers. Miss Ransome and her sister, who is also a deaconess, were among the members of the mission who were in Peking during the siege of 1900, and the former was the author of the graphic account of "The Siege Hospital in the British Legation," which attracted so much attention at the time it was published. For her conspicuous services to the sick and wounded throughout the siege, Miss Ransome was decorated by the King with the Royal Red Cross. Her splendid work at a period when her physical resources were most severely taxed, proves that she was endowed with some of the most essential of nursing qualifications.

### PRACTICAL COMPLIMENTS TO MATRONS.

Two retiring matrons have just received substantial evidence of the appreciation in which they are held. Recently, after some years' service at Crewkerne Hospital, Miss Cooper went to East Orange, New Jersey, U.S.A., and a banker's draft for £27 6s. was forwarded to her by the late secretary to the Hospital. Mrs. M. A. Tucker, on leaving Totnes Cottage Hospital, has been the recipient of a cheque for £24 3s., subscribed in recognition of her thirteen and a half years' work as nurse-matron.

### IRISH NURSES AND MASSAGE.

The Matron's Council of Dublin held their first meeting of the new session the other night at the rooms of the Irish Nurses' Association. The principal subject under discussion was the formation of a school of massage, with power to grant a certificate of its own. Miss Peter was among the visitors present. At present, though classes are held in Dublin by a London teacher who is a member of the Incorporated Society of Messeuses, candidates for examination are obliged to cross the water in order to obtain their certificate.

### BASINGSTOKE ISOLATION HOSPITAL.

Miss Annie Jenkins has resigned the post of matron of Basingstoke Isolation Hospital, to which she was lately appointed. Her action is due to differences which have arisen in respect to circumstances connected with the outbreak of typhoid fever in the borough.

### NURSING IN SMALL WORKHOUSES.

At the Poor-law conference held at Southport the Rev. Father Roche, of the Bucklow Union, read a paper on "Nursing in Small Workhouses and the Administration of Union Infirmaries." Among necessary reforms, he urged that the lives of the nursing staff should be made more tolerable, and their continued services encouraged by better food and accommodation, as well as by more recreation. He also dwelt upon the importance of the hospital, or infirmary, being kept for really sick cases requiring skilful nursing; and he considered it necessary to remind some Guardians that paupers and the sick poor are not criminals, and that they themselves are not prison officials. Wherever this spirit prevails there is not much hope of the nurses being treated as they ought to be. Guardians who regard the sick poor as criminals and themselves as prison officials are apt to imagine that so long as they pay the nurses their stipulated salaries they perform their duty. General agreement with the suggestions in the address was expressed.

### STUDENTS FOR THE SANITARY INSPECTORS' EXAMINATION.

An interesting series of lectures on Public and Private Hygiene, under the auspices of the National Health Society, is now being given at the lecture-room of the Society, 53 Berners Street. These lectures form part of the special training courses for lady students who wish to enter for the Sanitary Inspectors' Examination Board certificate, and for the diploma of the National Health Society. They are delivered on Tuesdays and Fridays at 2 p.m., and further particulars can be obtained from the Secretary, Miss Fay Lankester.

### OUR CHRISTMAS DISTRIBUTION.

Now that the holiday season is over, we hope to obtain every week many responses to our appeal for contributions to our Christmas distribution of articles of clothing for the use of patients in hospitals and infirmaries. We acknowledge with thanks a parcel from Nurse Mary Besant, 4 York Street Chambers, Bryanston Square, W., and a contribution from a reader at Horncastle, Lincolnshire, whose name is not enclosed. All parcels should be addressed to the Editor, 28 and 29 Southampton Street, Strand, London, W.C., with "Clothing Distribution" written on the outside.

### The Nursing Outlook.

"From magnanimity, all fear above;
From nobler recompense, above applause,
Which owes to man's short outlook all its charm."

### SHOULD HOSPITALS TRAIN PROBATIONERS WITHOUT PAYMENT?

The difficulties created by the continuous growth of hospital expenditure, especially in recent years, has engaged the earnest attention of the authorities of the great central funds, and of the managers of the best hospitals as well. The average cost under various heads of a nurse at eleven London hospitals is about £50 per head per annum, and at the provincial and Scotch hospitals about £45 per head per annum. Of course it is true that hospitals must have nurses, but there is no obligation on the hospital authorities to train probationers at all, or at least beyond the requirements of demand and supply so far as their nursing staff is concerned. Probationers and medical students who respectively aspire to become certificated trained nurses and medical practitioners have no claim as individuals to receive free education at the expense of the supporters of the voluntary hospitals. An analysis of the accounts of a large hospital soon shows the material effect which the present large expenditure upon the nursing department has had of late years in increasing the expenditure per bed. Apart from economical considerations and the nursing requirements of each hospital for the adequate tending of its own patients, every hospital committee would be justified in declining to give free education in nursing to any probationers who were not required for the work of the hospital. For our present purpose it will suffice for us to point out that here is an item in hospital expenditure which might be materially reduced by the abolition of free education, and that it has an important bearing upon the financial requirements of the hospitals, and the needs of the public in conjunction with the establishment of a private nursing staff in connection with each important hospital which would be adequate to meet the more pressing demands for nurses by private families.

Dr. Worcester, the initiator of the New Cambridge school of nursing in Massachusetts has recently given some interesting addresses on the advantages of the new school. This school originated in a petition which was unanimously presented to the trustees of the Cambridge Hospital to support a training-school of nurses like the Waltham School. The trustees were unwilling to undertake the responsibility. Dr. Worcester insists that the trustees were right, for it is not their duty to train nurses. The hospital might be driven to it if they could make no contract with some other institution to get their nurses, for it is the duty of hospital trustees to secure the best possible and the cheapest possible nursing service. The trustees of the Cambridge Hospital rejoined, however, that they could not afford the expense of a proper training school, and that they could not rightly undertake a second or third rate school, at any rate until it was proved that public spirited citizens would not establish a truly educational institution which might supply the hospital with a student nursing service. Dr. Worcester maintains that it would have been far better for the profession of nursing if other hospital boards had been as wise and conscientious as that at Cambridge. The effect of the action of these trustees has been that the smaller hospitals at Cambridge are reported to be all eager to secure a student-nursing service too.

It may be interesting to explain what Dr. Worcester's new school of nursing proposes to attempt. It will have to meet three needs. The pressing need of the Cambridge Hospital and of the smaller hospitals. The need of the district visiting, and the home needs of the people where graduate service cannot be afforded. At one point Dr. Worcester is at issue with many nursing reformers in the United States. He maintains that the student-nursing service, by supplying these needs will render an all round and complete training at last possible, and that it is wrong to claim that this result can be attained by nurses who have been trained only in hospital service. Probably the majority of those of the training authorities in this country whose opinion carries weight will be inclined to agree with Dr. Worcester, realising as they do, that under the present system no hospital can afford complete training in all branches of nursing to its pupils. Dr. Worcester's argument is based upon the contention that, although for hospital nursing only such education is needed as can be acquired in the wards, it does not at all follow that such an education fits the nurse for home nursing. In fact it is notorious that it does not do so, yet it is fitness for home nursing and not merely for hospital nursing, Dr. Worcester urges, that ought to be the objective of all training schools of nurses, for it is in home nursing that nine out of ten nurses undertake to practise their profession. Again Dr. Worcester thinks that no hospital ever should undertake either a nursing or a medical school; a truly educational institution can never rightly be subordinated to an eleemosynary institution, hence, the only hope of educational advance in the teaching and training of nurses lies, as Dr. Worcester contends in the co-operation of hospitals with independently established schools of nursing. The school of nursing would be so organised as to enforce the threefold necessity for all student nurses, (1) of a preparatory course, (2) of a thorough training and drill in hospital nursing and (3) of teaching and training in home nursing. The first two courses can be secured by proper organisation, but the last is a novelty in this country and it may be interesting to explain how Dr. Worcester proposes to meet it. It appears that the medical practitioners and many families in Cambridge and Waltham, Mass., have been accustomed for twenty years to employ the work of student nurses. Such service affords the very best educational facilities through district visiting nursing, and can only be supplied by the student nurse. Seeing that the plan has succeeded and is popular in Massachusetts there is good reason for giving it a trial. Dr. Worcester insists, in support of his view, that it is as economically wrong to depend upon graduate nurse service in district visiting work as it would be for hospitals to depend upon graduate nurses.

### Abdominal Surgery.

By Harold Burrows, M.B., F.R.C.S., Assistant Surgeon to the Bolingbroke Hospital.

### I. Introduction.

It is nearly true to say that there was no such practice as abdominal surgery thirty years ago. Until Lister showed how wound infection might be avoided the dangers from sepsis were so great that none but the hardiest surgeon would venture to open the peritoneal cavity. A few attempts were made from time to time, but the patients died almost invariably. Circumstances are very different now, and the results of abdominal surgery are excellent; but no finality has been reached, and the results show signs of improvement every year. This improvement is not due entirely to better surgery, for better nursing has had its share in the progress and may claim its proportion of the credit.

So important is the part which the nurse has to take in abdominal surgery that no surgeon would think of undertaking a serious operation case, unless compelled by dire necessity to do so, in the absence of skilled and intelligent nursing. For on this will rest the result to a considerable extent. The best nurse is one who combines knowledge with self-devotion, and sympathy with cheerfulness. It is necessary to emphasise this because a clever, well-educated nurse sometimes happens to have a very bad bedside manner, which more than half counteracts the advantages of her technical knowledge. Sick people are very amenable to the subtle forces of suggestion, and they often reflect in a remarkable manner the mental attitude of those near them. So if the nurse is moody and impatient the invalid will be moody and impatient too; if the nurse is cheerful and bright her charge in sure to be in tune with her, and his chances of getting well will be all the better. There is another point. If a patient is allowed to feel neglected, if his little desires and requests seem to fall on inattentive ears, he is extremely likely after a time to become sulky, and to refrain from asking for help when he really needs it; which is a very serious matter.

But, important though this is, the essential qualities of a nurse neither begin nor end with the possession of a proper temperament. Her duties require in addition considerable technical knowledge if she is to be the intelligent helpmate of the surgeon. This knowledge is only to be acquired by experience and by constant application and readiness to gather technical information at all times. A nurse should be acquainted with the chief functions of the abdominal viscera and the manner in which these functions are performed, and she should have a sound working knowledge of the structure and relative positions of the organs, in order to appreciate the requirements of abdominal surgery.

### Anatomy.

It is not intended to discuss fully the anatomy and physiology of the abdomen in these pages. But a rough survey of the general anatomy of this part of the body will be given, chiefly for purposes of reference later on.

The abdomen is the space which lies between the chest and the floor of the pelvis. It encloses the greater part of the digestive organs, the spleen, great omentum, and portions of the genital and urinary systems. The cavity is limited above by the diaphragm, which separates the abdomen from the thorax, the lower boundary is the floor of the pelvis, and the walls are the vertebral column behind, and the abdominal muscles at the sides and in front. The cavity is lined by a smooth, glistening membrane called the peritoneum, which also forms a covering for the abdominal viscera. It is not easy to convey a satisfactory impression of the peritoneum by verbal description, even with the help of illustrations, but a good working idea of its structure and arrangement can be gained in a few minutes by demonstration on the body of a dead animal, for example, a rabbit. It will be well worth the while of any nurse who is interested in her profession to ask some medical friend to give her such a demonstration. In the meantime some notion of the arrangement of the peritoneum and the manner in which it covers the abdominal organs may be obtained by referring to the diagram given below (fig. 1) which represents a transverse section of the abdomen at the level of the umbilicus.

Fig. 1.

A. Parietal peritoneum. B. Visceral peritoneum. C. Mesentery (consisting of two layers of peritoneum). D. Small intestine. E. Ascending colon. F. Descending colon.

The peritoneum lining the abdominal wall is described as the parietal layer, and that which covers the abdominal organs is the visceral layer.

It is curious that while the parietal peritoneum is extremely sensitive to injury the visceral layer is not at all sensitive in this respect. This fact should be remembered; it will be referred to more than once in the course of subsequent articles.

Abdominal surgery might well have been termed peritoneal surgery, for the difficulty of avoiding septic peritonitis (inflammation of the peritoneum) is the chief embarrassment to the surgeon. Indeed, for practical purposes the limits of the abdomen are those of the peritoneum.

### Areas of the Abdomen.

For convenience of description the abdomen is divided into certain arbitrary divisions. The part lying below the level of the brim of the pelvis is known as the pelvic portion, or simply as the pelvis. The remainder is divided into nine regious by imaginary planes (see fig. 2). The middle regions (1, 2, and 3) are named respectively from above downwards the epigastric, umbilical, and hypogastric. The

Fig. 2.

lateral regions, from above downwards, are named the right or left hypochondriac, lumbar, and iliac.

The chief contents of these regions are as follow :—In the epigastric region are the left lobe of the liver, part of the stomach with its cardiac and pyloric orifices, the bile duct, part of the duodenum, the pancreas, and the solar plexus.

The right hypochondriac region is chiefly occupied by the right lobe of the liver; it also contains the gall-bladder, the upper end of the right kidney, and parts of the duodenum and large intestine. The left hypochondrium contains a portion of the stomach, the spleen, the upper end of the left kidney, a portion of the larger intestine, and sometimes the tip of the left lobe of the liver.

The umbilical region contains the great omentum, the transverse colon, the greater part of the small intestine and the mesentery.

The lumbar regions contain some convolutions of the small intestine, portions of the large intestine, and the kidneys and ureters.

In the hypogastric region are found the lower part of the great omentum, coils of small intestine, the uterus when it is enlarged, and the bladder when it is distended.

The right iliac region contains the cæcum and the appendix, and the left iliac region contains the sigmoid loop of the large intestine.

In the pelvis are the bladder, the lower parts of the ureters, the rectum, and some coils of small intestine; and in the female the uterus and its appendages (broad ligaments, Fallopian tubes, and ovaries).

### The Nurses' Clinic.

### NURSING HEART CASES.

There are not many diseases in which the nurse can do more to make her patient's life bearable than in bad heart cases; so much depends on the way in which he is generally cared for; and yet to nurse him satisfactorily will often tax her ingenuity to the utmost. Take, for example, such a patient as we continually see arriving in any medical ward. He is generally in a panting, breathless state, suffering from the necessary exertions he has made in getting to the hospital, cold, and blue. Clearly, the thing to be done is to get him to bed as soon as may be; but she must not be in too great a hurry. As the motto before the eyes of the 'bus driver is "Drive slowly," so the motto over every heart patient's bed might be "Nurse me slowly!" Whether in washing, feeding, or moving, he must not be hurried. Even more important than getting him to bed is letting him sit quietly by the fire for a few minutes while he gets a little breath for the business of undressing. And he will be none the worse for a little hot food while he is waiting.

He need not have a thermometer thrust into his mouth almost before he realises that he has arrived at his journey's end; he probably is obliged to breathe with his mouth open, or if not, he will feel obliged to hold the thermometer in, which is an exertion he ought not to be called on to make. The treatment that will do him most good is the thorough rest that will be ordered for him, and this should begin from the time he enters the ward.

Very likely a leisurely warm bath may relieve him; but this, of course, is not to be given without the doctor's permission.

While he is waiting there is his bed to think about. Extra pillows must be got, a hot bottle ready, for with bad circulation his feet are naturally cold. His clothes must be loose and warm, flannel for preference, and made so that there will be no struggles to get the shirt over his head for the doctor's examination. In making the bed the blanket should come up higher at the top than usual, so that it may draw up well over the chest.

The pillows will need rearrangement when the patient is in bed, else they will probably be as comfortable as an average railway carriage. The necessary support can be given by making them even from end to end, so that on lying back he sinks into them a little. The top pillow should be nearly on his shoulders, and arranged to keep his head from falling over to either side. No "born nurse" can resist the feeling of gratification that comes over her when she hears the "That's better" which will most likely take the place of a "Thank you" for her efforts!

This sitting attitude is instinctively adopted because the patient feels that it eases his breathing, the weight of the abdominal organs being kept off the heart. Of course it is not a restful position, but too often it is the only one possible. In this position, too, the fluid of the body tends to collect about the loins and buttocks, and it is anxious work to prevent bedsores. The skin gets half sodden, and it is out of the question for the patient to stay on his side more than for the shortest possible time while the gentle friction is applied that does so much in keeping backs healthy. The only thing to be done is to begin from the first to try to harden the skin with spirit, and to take special care in using only soft old linen for draw-sheets.

Rest in bed has a wonderful effect on heart cases. It is not much use to keep a patient in bed if he is allowed to walk to the lavatory, for a very little exertion soon undoes all the good he is getting by weeks of bed. A deaf ear must be turned to all entreaties of the kind, and absolute bed enforced.

His food must be "light and nourishing." It may be asked, "To whom would you give food that was heavy and not nourishing?" But the heart case needs lighter food than other people. Fish, game, and poultry are better for him than coarser meat, and such things as potatoes and cheese should be taken in strict moderation. He must be fed in small quantities, and oftener than is usual. It is most important that the heart shall not be hampered by a full stomach pressing against it. Many a man has died from heart disease through going to bed directly after eating a large supper. A rather dry diet, too, is frequently desirable. The question of alcohol belongs to the doctor.

His appetite is capricious. If he fancies his food at odd hours—*e.g.* tea and toast at 3 A.M.—he had better eat it then than wait till breakfast time and find his appetite gone. Distressing sickness, retching and nausea often accompany heart disease, and are very serious signs of increasing illness. Besides the loss of nourishment if sufficient food cannot be taken, the strain of retching adds greatly to the heart's work. It is important, therefore, to prevent sickness by all the means we can. Food must not be pressed, for its rejection shows that the stomach is not in a fit state to digest it. A few spoonfuls of milk and barley water kept down, will do more good than a pint returned. Sometimes sips of very hot water give relief. Flatulence is a troublesome, and, indeed, dangerous symptom; a stomach and abdomen distended with gas press as hardly on the heart as a stomach overloaded with food. Here the nurse can come to the rescue with hot peppermint water, or a few drops of essence of ginger in hot water; and very hot cotton-wool, often changed, has some effect in getting rid of it.

**THE NURSES' CLINIC**—*Continued.*

Sleep is a difficulty with these cases. Yet, if it is remembered that the heart's beats are about ten less in a minute during sleep, it is seen how necessary it is that this period of partial rest should be as long as possible. Besides getting the patient into a comfortable position, and seeing that his last meal is a light one, it will be found that he sleeps better if he has fresh air in the room. A draught must be guarded against, for a cold is serious to such a patient. "It is better to have a fire and an open window than no fire and a closed window." If pain prevents sleep a poultice with a little mustard in it may give relief, or even a plain hot flannel.

The bowels must be kept well open, partly with the object of getting rid of some of the fluid that is choking him up, and even more for the sake of preventing straining in defecation. It cannot be impressed on the nurse too strongly that every effort the patient makes gives the weak heart extra work, which may result in its stopping work altogether. Straining, retching, and coughing are extremely hurtful.

There is often some delirium at night. The worst thing that a nurse can do then is to struggle with the patient to keep him in bed. She must use only her wits and what moral control she has over him, trying to turn his mind on some other subject than getting up.

### The Nurses of Grimsby Hospital.

INTERVIEW WITH THE MATRON. BY OUR COMMISSIONER.

The workmen were still in possession when I visited Grimsby and District Hospital one glorious morning in September, after a drive round the town had enlightened me as to its characteristics, its needs, and its colossal industry. The addition to the handsome building—which stands in a singularly pleasant and convenient situation—now in course of construction, is the convalescent wing, consisting of two wards with 14 beds, the gift of the Grimsby Amalgamated Friendly Societies, and one of many practical evidences of the affection of the workers for the institution. I heard from the Matron, Miss Frances Crichton, under whose

Grimsby Hospital.

auspices I went over the hospital, of many things to attest its popularity. We started—after a preliminary chat in her pretty room—with the old part of the Nurses' Home, which is an integral portion of the hospital, discussing the nursing and other arrangements as we proceeded.

"These are the quarters of the night nurses," said the Matron as we looked into one of several comfortable rooms. "And each night nurse has a separate bedroom."

### On and Off Duty.

"How many does the night-staff comprise?" I inquired.

"Three and a night sister. They go on duty at 9 p.m. and are off at 8 a.m. They have two hours off duty every day, and they are required to go out for an hour each day. This also applies to the rest of the staff."

"When do the day nurses come on duty?"

"At 7 a.m. They are off at 9 p.m., the wards all being closed at 8. All the nurses get half-a-day off once a fortnight, besides the time mentioned on Sunday; and I am trying to arrange a whole day occasionally when they can be spared. On Sunday half the staff are away in the morning from 9 to 1, and the other half from 2 to 9.30. The nurses have an annual holiday of a fortnight the two first years and three weeks the third year. Sisters also have two hours off duty daily, a day off every fortnight, and a month's holiday. There are ten nurses in different stages of probation and three sisters. The Matron acts as home sister.

We had reached a charming corridor with windows so

Nurses' Sitting-room at Grimsby Hospital.

constructed that they can be easily kept clean as well as open, and as we crossed it the Matron remarked:

### The New Bedrooms.

"Now we come to the portion of the Home which we owe to The Hospital. I mean the separate bedrooms which, at the Editor's suggestion, we substituted for cubicles.

"The bedrooms, I see, are similar to those in the old portion. You prefer these to cubicles?"

"Everyone in the hospital, from the Chairman downwards, is delighted that we did not have the cubicles. The nurses greatly appreciate the privacy of their own rooms."

"They are very nice," I rejoined as I looked at several of the tastefully decorated little rooms, the linoleum on the floor matching the furniture, additional air coming through ventilators over the door, heat from pipes below, and illuminated at night by the electric light.

"Neatness, cleanliness, and comfort are kept in view.

No boxes are allowed in the rooms, nothing is put under the beds. There is a strip of carpet in the centre of the linoleum. Where there is no room for a wardrobe to stand by itself it has been fitted in."

"Not only are the nurses' rooms very comfortable, but you house your domestic staff remarkably well."

"Yes; I do not think that wardmaids and servants could fare better. In the new part of the Home you also see that a fire-escape, to which admission is gained by three windows, has been placed close to the bedrooms. The lavatories and bathrooms are very handsome, with the most modern fittings.

The Theatre, Grimsby Hospital.

"The whole of the additions, including even the paper and the colouring, seems to have been most successfully carried through. I presume that there are no sitting-rooms in the home?"

Gifts to the Nurses' Sitting-room.

"No; the nurses' dining-room and sitting-room are both in the hospital."

The former was the first room which we entered in the main building. It is also at present used as a committee-room, and here on the walls are portraits of Mr. C. F. Carter, the present Chairman—presented at the opening of the Out-patients' Department in August—Mr. Broadhead, founder of the hospital, Mr. Veal, and other prominent friends. These will be transferred, in time, to the new Board-room. In the cheerful sitting-room the Matron mentioned that all the contents were presented.

"The piano, a very fine instrument, was purchased from the sums paid for admission to sports got up by H.M.S. *Alarm*; the carpet and the writing-table were bought with the proceeds of two whist-drives; the pictures were given by a member of the Committee; the comfortable arm-chairs and the overmantel were individual gifts.

The Women's Ward.

Passing three small rooms for private patients and the sisters' sitting-room, we walked through the women's ward. Here I noticed a piano, and the Matron said:

"There is an instrument in every ward. A service is held in each once a week, and, thanks to the good offices of a member of the Committee, there is singing every Sunday evening for half an hour in each ward when the patients are well enough.

"Have you more male than female patients?"

"Yes, there are only 13 beds for women; ten in this ward and three outside. We get a large number of abdominal cases, many of gastric ulcers, treated medically and surgically, severe accidents, such as a bad compound fracture of the nose and compound fracture of arm, which you will see in one of the male wards. And we have also just now a little girl in a private ward suffering from typhoid."

In every ward are patent ventilators, by means of which air is obtained without draught, an up-to-date bathroom, with Doulton basin, a sluice sink, tiled walls, and a shute which is used for conveying the soiled linen to the bottom of the building, so that none of it is left about for a minute. Here, and also in the Yarborough Ward—called after Lord Yarborough, the President—were a test-table and a steriliser, as well as flowers in abundance.

"The doctor likes the sister to do the testing," continued the Matron. "In fact there is a good deal of experience to be gained here by nurses which could not be gained in a hospital with a school, or in a larger institution."

"It is not often that larger institutions have such spotless corridors."

"They are all washed every day. You see in an institution like this the Matron is able to be sure that her instructions are observed. To each maid I give a written list of her work for the week, this never varies ,so she knows what she has to do every day.

"And the Matron's duties are more varied?"

"Well, they extend to the smallest details. For example, there is no porter's lodge, and the Matron has charge of the dressings. The Matron also has to attend to outside requirements. We always keep a good supply of oxygen, and if anyone in the town appeals to us for oxygen we let them have it."

In the Theatre.

"Do you get many operations?" I inquired, as passing through the anæsthetic room we approached the perfectly-appointed theatre, with its very modern table.

"Yes, a large number. The medical staff operate twice a week, as well as in emergency cases at night. Each nurse has three months in the theatre. The head sister has always the assistance of two nurses at an operation. We are just about to spend £20 on new instruments, and no reasonable expense has been spared upon the theatre."

The Children's Ward, Grimsby Hospital.

"The training, of course, includes lectures from the honorary staff?"

"Yes; on all subjects. At the end of the three years the nurses have to pass an examination given by one of the honorary staff, and if they succeed they receive a certificate."

### THE NURSES OF GRIMBSY HOSPITAL—*continued.*

Having glanced into the *x*-ray room, where absolute darkness is ensured, we went into the Veal Ward, containing ten beds. Several of the patients were foreigners, and I learnt that the foreign element includes Dutch, Swedes, Norwegians, Germans. A Swede suffering greatly from rheumatism had just been brought in. More interesting still was the Princess May Children's Ward, with its 12 beds, three swing-cots, and pretty stove. This was furnished and fitted by the Working-men's Committee, and though some of the children looked exceedingly ill, they were all bright and happy.

### The Out-Patients.

"Now," said the Matron, as we made our way downstairs, "I will show you the new out-patient department which enabled us to get the Nurses' Home. It is spacious and convenient, and has one examination-room for men and another for women. These rooms were badly wanted. Also the far superior accommodation we now possess. The number of out-patients is very large. I suppose we get about 50 cases on Tuesdays and Fridays, as well as about 30 casualties every morning. A sister is in charge."

From the out-patient department we crossed the yard to the laundry, the mortuary, and the dissecting-room, and returned to the well-kept kitchen and commodious stores.

"You appear to be well off for vegetables," I observed.

"The harvest festivals all round the country stock us with sufficient to last until the beginning of the year; ice is supplied to us *ad lib.*, free of charge; and a firm in the town sends lemonade for the nurses to have twice a week."

### Recreation and Pay.

"What about recreations for the nurses, in addition to their charming tennis and croquet ground?"

"They are rather fortunate in that respect. The father of one of the medical staff sends them tickets for sails; the railway company sends them tickets to Cleethorpes; Lord Yarborough supplies papers; and concert and theatre tickets are often forwarded. Most of them cycle, as the roads are beautifully level. I never refuse to give them late leave; but they do not ask more frequently than about once a month."

"Do you receive plenty of applications for vacancies?"

"Yes; there is never any difficulty in filling them. Last year's Committee granted a salary of £6 for the first year; for the second the salary is £10; and £15 for the third. Indoor uniform is given, but outdoor uniform is optional, and has to be provided by the nurse. The sisters receive £30 a year. When we recently advertised for a sister we had 14 good applications."

### Miss Stewart's Influence.

"How long have you been Matron here?"

"A little over two years. I was trained at St. Batholomew's Hospital, and owe much to Miss Stewart. Indeed, but for her I should not have continued my nursing career. I finished my course and remained on as staff nurse in different wards for more than two years. In the absence of sisters I had charge. Then I went to the Royal Naval Hospital at Haslar as sister for three years. I returned to civil work, and was night sister for four months, and home sister for six months at Birmingham General Hospital. In December, 1902, wishing to be proficient in housekeeping, I became Assistant Matron at the City Hospital, Lodge Moor, Sheffield. There I had control of the housekeeping and store department, as well as the nurses' and the maids' "homes."

"And at Grimsby the re-furnishing and re-modelling of the hospital has been carried out under your supervision?"

"I must leave others to speak of my work here; but I have found it congenial and have always enjoyed the sympathy and support of the governing body and the medical staff."

### Casualty Hour.

"Really time cheats!" you mentally ejaculate as you hear tramp, tramp, pitter patter, trip, trip along the stone passage, and you know the surgery is filling up, for it only wants ten minutes to 9.30—the casualty hour. Someone wants a drink, a pillow altered, a bandage eased, a handkerchief picked up, a letter found. Sister, who ought to know better, wants a lot of things, and thus ten minutes dwindle into one ere you change your apron, take a breath, pat your hair, and go forth to see that all is in readiness. The scene at first sight is quite cheerful. The surgery is a good sized room. A large window occupies the centre. Cheery fire, brightly draped screen, and substantial couch one end. A white tiled table on which stand glass boxes with silver-plated lids resplendent, their contents (cyanide and iodoform gauze, blue wool, and pink boracic lint) making a rainbow of soft tints. A long shelf over a glass dressing table displays a row of lotion bottles, equally varied in colour, and boldly labelled in deep-set flaming letters of red. The silver plated boiler and sterilizer hum reassuringly, while the spotless white tiled sink and basin, brilliant taps, shining glass jars, and instruments all loudly proclaim that the hand of the faithful, and doubtless weary, probationer has neither faltered nor failed this morning.

No one would imagine that this is a chamber of torture.

Now the familiar flip flap of oversized but otherwise irreproachable slippers worn by the assistant house-surgeon greets your ear, next his voice.

"Good morning, nurse."

"Good morning, Mr. Bell."

"Now then," he says, turning to the patients, "look sharp. Names, please. Old cases first."

The register is rapidly gone through, new names entered,

The Latest Thing in Probationers.

and work begins in earnest. The bench is full, and as each patient is seen another takes his place.

Mr. Bell directs, and three nurses work as hard as they can, syringing, fomenting, bandaging.

"Now then, Tommy, what's the matter with you?" A small grimy urchin holds up a still more grimy finger with a fish hook firmly attached. A slight sound follows, a sigh, a whimper, and the child goes out knuckles in eyes.

The Patient is Assigned to a Vacant Bed.

An old woman presents an apparently older ankle swollen with rheumatism, and with due deference to her years is requested to take a seat near the fire, to await the house physician's verdict.

"Bide a while till another young man comes along?" she asks. "Certainly, sir," and a broad grin spreads over the bench.

Then a man puts forth a septic hand. There is a slight debate, then, "Very good, sir," the flash of a knife, you are told to apply a boracic fomentation, and with a civil "Thank you, nurse," the man goes on his way.

Next. "Just hold this leg a minute, please, nurse." Needs must, so you go, but turn your face from the reproachful eyes of a child while the surgeon probes. "Suture, please. Just steady this lad's head." It is a tough scalp, but yields beneath a firm hand and sharp needle. So—prick, swab, knot—a schoolboy's lusty yell and a dry dressing complete the operation.

Now comes another septic hand. This time it belongs to a maidservant, piteously anæmic and shaking dreadfully. There follows the hiss of the ether spray, a groan, a succession of nervous ohs! You put on a chlorinated soda fomentation hastily, while someone gets water and presses her head on her knees just in time.

A mother presents a fretful baby with a large lump behind the right ear, and your heart sinks as you hear her refusing to let it be admitted.

A young woman, pale and sad-eyed, removes a shawl and discovers shoulders cruelly bruised and blackened. The surgeon on coming utters a low whistle. "I fell downstairs, sir," she says with averted eyes, and your own drop as you realise the obvious untruth is in self-respect.

"Lead and opium compress, nurse." "That," he explains kindly to the patient, "will ease the pain." Then, in an inaudible aside, "And may some scoundrel get the jolly good thrashing he deserves."

Now the slow laboured tread of men who carry a burden reaches us. One end of the surgery is quickly cleared and screened, to admit—

"What is it, Brown?"

"Fractured famor, sor."

Brown is initiated, being a first-aid porter. The hum of minor ills sinks and dies away like a frivolous breeze before a great storm. Their treatment is suspended for a few moments with glad assent, for the poor are nothing if not sympathetic to each other. The latest thing in probationers hastens to fill hot bottles; the staff nurse, after a glance at the length of the fractured limb, produces a long "Liston," wool, bandages, binder, strapping, pins, pad, etc. The house-surgeon arrives, and ere long the limb is set, splint fixed, and the patient assigned to a vacant bed. The gasping audience on the further side of the screen begin to breath normally again.

Then comes tap, tap of a crutch, and you take heart at the sight of a sunny face. "Good morning, sir. Good morning nurse. My leg," with a glance at the offending limb, "is going on fust rate. I'll soon be in the team again playing forrards." "That's right, my boy," says the surgeon, while he examines the leg. "Splendid! You do us credit, doesn't he, nurse?" A flash of teeth and "Thank yer, sir. Good-day, sir," and tap, tap goes cheerfully down the corridor again. But it returns a trifle less imperative this time, looking up you find a waving arm outside the door, beseeching your help. You hasten to render assistance, and simultaneously the arm disappears into a ragged pocket and out comes a bunch of violets. "Please 'ave 'em, nuss; they're fresh as dew."

You bury your nose in them, smiling at the ruse. "Oh, thank you. How lovely!" But tap-tap is half way down the

You Take Heart at the Sight of a Sunny Face.

You Hastily Finish a Fancy Head-dress.

passage again, for the sons of the people share with the sons of fortune a sense of chivalry, and you are left to "divine amends for a courtesy not returned."

"Dry dressing and a bandage wanted here, nurse, please." So you hastily finish a fancy head-dress and find yourself confronted with the graceful, if conventional, curves of a "capeline."

**CASUALTY HOUR**—*continued.*

Later you glance up from the bathing of a crushed foot to see what manner of child the surgeon is promising a "silver penny," a just reward for a "good little girl." Hum!—a pretty tearful face; so the surgeon has weaknesses, too, has he? A shadow falls across the door and a student with a nervous air hesitates on the threshold. Mr. Bell looks up. "Come in, man! Come in! There's plenty to do. You might get that plaster case off, will you?"

With saw in hand the student approaches a boy whose leg is in a plaster of Paris case. But no one is quicker than a child to detect an uncertain air.

"No! no!" he cries, with protesting hands outspread. "You'll cut me leg off, I know you will. Oh! oh! Mother! mother!"

The poor dresser flushes painfully, and, with admirable patience but dejected air, suggests that he shall make a start, but will stop at the first warning that the saw feels near the skin.

Oh, hapless bargain!

All goes well for a few moments. The patient's sighs relieve the monotony of the saws. The dresser pauses to mop his brow and—is lost. On recommencing the patient rebels.

"Oh! oh!"—frightful yells. "Ain't you done? That's me leg! You've cut it! I knew yer would—I told yer so.

The Hospital Cat Seats Herself on the Dresser's Chair.

Oh! oh! oh!" The dresser stops in pained alarm and looks appealingly at Mr. Bell, who strides over and glances at the leg.

"Now then, stop that noise this minute," he says to the patient; and, "Get on, man; you're not through the plaster yet," to the dresser.

In contrast a man sits with set white face while you dress a ghastly burn, causing doubtless at every touch exquisite pain. When at last it is finished his "Thank you kindly, nurse," is free from any note of bitterness and you wash your hands somewhat solemnly, perhaps, pondering for the several hundredth time over the unanswerable but eternal why?

"When you've quite done washing your hands, nurse, perhaps——"

"Yes, yes, sister," you hasten to reply, and then comes a grand clearing up. A merry, if quiet, scrubbing of bowls and instruments, and more cleansing of hands. What a pity we can't sterilise them straight off and have done with it.

The room is fairly clear when the hospital cat walks in, and, having sniffed round, condescendingly seats herself on the dresser's chair with a solemn blink at the surgeon, as one who would say:

"You won't try your skill on me, young man. I've come here for a nap."

"Shoo," says the surgeon, "just look at that impertinent cat. Well, I'm off to breakfast."

"Haven't you had it, Mr. Bell," you say for the seventh time that week, but you do endeavour to express sympathetic surprise in your tone.

Here the clock hastens to remind you once again that time gallops and that ten minutes is not too long in which to get lunch, letters, and a glimpse at the papers. So off you go and forget, pro tem., the sorrows of the world.

"So passes all confusedly.
  As lights that hurry, shapes that flee,
About some brink we dimly see
  The trivial, great,
Squalid, majestic tragedy
  Of human fate."

### A Visit to a Ceylon Hospital.

BY A NURSE.

I left the Terminus Station, Colombo, by the night mail and settled myself down to sleep. At midnight the difference in temperature was perceptible as the hot plains were left rapidly behind, and it was with a sigh of relief that I curled myself more cozily under my rug and said "Good-bye" to mosquitoes and hot, stifling weather.

When I woke up we were nearing Hatton, and the view was glorious. Grey mist covered everything at first, and then, little by little, the earth coyly peeped out and the sun glittered suddenly on the whole. Blue-grey hills, their tops still hidden by the mist, the tender green of the paddy fields making such a rich contrast with the more sober shade of the tea plantations which cover all the hill slopes. It was the most beautiful scenery I had ever seen. As we got higher and higher we came across foaming cascades and tumbling, tossing hill streams, with tiny bridges. At 8 A.M. we reached Nanu-Oya. My friends were waiting for me with a carriage. It was cold and almost too bracing at first. An hour's drive up the hills brought us to Newara Eliya, the pretty sanatorium of Ceylon. The houses are widely scattered among the hills and around the lakes. There is a nice racecourse and a gymkhana, and near King's Cottage stands the Governor's residence. There are also splendid golf links. In the afternoon I visited the hospital, situated some way out of Newara Eliya, nestling at the foot of a hill. There is a nice little garden leading to the entrance, then a dispensary, the male medical and surgical wards, and the nurses' quarters, forming the main block. The building is low. The wards are dark and not very cheery looking. The nursing staff consists of an untrained matron and a trained nurse, the latter really doing all the responsible work.

The nurses' quarters are very disappointing. A tiny room serves as dining and sitting room, and would have been very bare and comfortless had it not been for photographs, etc., scattered about. The bedroom—dark, small, and damp—struck me as the most cheerless place for two tired nurses. From there I was taken to the female block. We passed up the hill, through a very pretty garden, but during the rains there is no shelter for the nurse, continually obliged to go up and down. The female wards consist of a combined surgical and medical ward, and separate from it the female diarrhœa ward. There was plenty of light here, but the arrangements are very unsatisfactory. This is always crowded owing to dysentery being constantly prevalent. It contains for all furniture waxed wooden beds, with a pillow, mat, and blanket for each patient. In the middle of the bed a circular hole is cut out, under which is placed a tarred wooden bucket, to avoid the use of the slipper. Added to the general overcrowding in this ward, such a method, it can be easily imagined, is most unsanitary.

I next visited the Baker Memorial Ward for the use of Ceylon tea-planters. This is prettily situated farther upthe hill. There are there large, airy, single wards, with dressing and bath room attached, a nice dining room, a nurses' bedroom, and a kitchen. Surrounded with flowers, up near the clouds, away from noise and din, with a willing, intelligent nurse to attend to their wants, the Ceylon planters have an ideal spot for being by care brought back to health.

### Incidents in a Nurse's Life.

### THE LIFE OF A NIGHT.

The electric light was turned low, and the red wire gave but a dim light in the quiet ward. Four figures lay stretched out in attitudes suggestive of exhaustion or repose, and in the little cots by each bed lay a tiny spark of new life. A nurse stood by one of the beds; it was the last "case," and the newly-made mother was but just settled and at rest; but it was the little one in the cot who held her attention, by its ineffectual hold on life, and caused the anxious look upon her face.

A half-checked sob came from the mother. "Nurse" turned and spoke almost sharply, she was tired and not in any mood to sympathise with "hysteria."

"Come, Ellen," she said, "you must really keep quiet and go to sleep; you are all right now, you know, and have nothing to worry about at all." Oh, the thoughtless words one often speaks! "Nothing to worry about." Poor mother—alone, uncared for, and unloved at the supremest moment of a woman's life, and with a little unwanted and unwished for life by her side which should have been an unmixed joy and comfort. But Nurse was tired and one case is so much like another, though perhaps if she had been less "rushed" she would have seen the pathos of such a life even on the surface, and might have felt the irony of her thoughtless words. As it was she turned impatiently to the cot once more, and as she did so a feeble wail disturbed the silence of the ward, and the mother, who had turned her face to the wall when Nurse had spoken, once more moved uneasily and opened her eyes. "Oh, do let me have him while he wants me!" she sobbed. "He won't want me long; but I'm glad it's a boy, boys do things, and people only ask what they do, not what they are, so he may forget me. I am glad, but let me have him while he wants me. With girls it's different; I should have always had her. I have got nobody else left now. But I'm glad it's a boy. I hope I shall die as soon as he can take care of himself, then he will be easily able to forget me. Do let me have him."

Remembering that the doctor would not "do his round" for an hour or more, though against the rules, Nurse, unwillingly softened, took the tiny little thing from its cot, where it seemed so lonely, and put it against its mother. The plaintive little cry stopped. The mother, holding him close against her, bent her head to let her cheek touch so gently the baby's head, and her lips generally so sad, smiled, the faintest murmur escaping from them seeming born of an almost sacred breath of contentment. It passed through the nurse's mind "why was this rare sweetness which all the wild young enjoy denied this little creature?" "Because the mother, being human, was not credited with taking sufficient care of it?" "Then civilisation must be going in the wrong direction; we should get more happiness by turning it back." But this train of thought was becoming too complicated for the nurse's busy brain and another patient was now requiring her attention.

When at last she came to remove the sleeping child the mother clasped it convulsively in her sleep and sighed, but worn-out nature had its way and she sank again to rest.

Looking on the face, pale and worn, but with lines of refinement and delicate beauty, the Nurse wondered much what the past life had been which had now come to such a tragic climax. An hour passed, the patients were all asleep, the doctor had done his round, and beyond keeping on the alert for the slightest sound Nurse had nothing to do in the ward for a short time. She walked wearily into the "duty room" and sank into the only chair. Glancing at a small dish in which were two rashers of uncooked bacon, the top of a cottage loaf, and a lump of butter, she felt that the solitary meal would not be worth the trouble of preparing. Pulling the chair in front of the fire, which really was comforting, with her elbows on her knees, she rested her head in both hands, and let the heavy swollen eyelids fall over the burning eyes "just for a moment." She was at home sitting by the fire in her mother's bedroom. Then with a frightened start she sprang up, angry with herself. How long had she been sitting there? She listened intently for any sound from the ward, then glancing at the clock she found that it was only three minutes since she came into the duty-room. Feeling relieved and somewhat roused she put the kettle over the fire for making tea.

A few minutes later life seemed rosier, and Nurse's thoughts travelled back again to the new baby and its mother. How could one so utterly unselfish in her absorbing pure love for her child be commonly so accused and outcast, because she had followed not the customs of the land? And what could have led her to an action meaning so much wretchedness? Perhaps her environment, through poverty, had been so degrading that she had lost all self-respect and did not realise or did not care what the world would think of her. Or perhaps she was a simple country girl, who, too, had a mother away in some country town or village who loved her, even as she loved her babe, and who would have cared for her as tenderly could she have done so. But the girl, full of wild, tangled dreams of a freedom which she herself could not understand, burst one day through the monotony and was carried away by the restless current of the age out of reach of her mother, and perhaps later out of her depth. At first there came news of exciting enjoyments and friends; but later the mother had waited for each post time in vain, not daring to own even to herself with what longing and anxiety, hoping on from day to day and never losing faith in her child. Perhaps—perhaps—but time was passing, and Nurse roused herself and shook off the reveries which absorbed her, turning once more to the duties before her.

. . . . . . . . . . . .

As the cold grey of early morning fought and conquered the warmer light in the ward, a greyness more enduring than that reflected upon it from without, dawned upon the baby face. The tiny breathings, which seemed no more than the light summer air stirring the petals of a flower at evening, died away and an absolute repose filled the little fragment of humanity. A pebble cast up for one moment on the shore of time by the ocean of eternity, to be the next withdrawn again into its own vast and incomprehensible bosom.

### To Nurses.

**We invite contributions from any of our readers, and shall be glad to pay for "Notes on News from the Nursing World," or for articles describing nursing experiences at home or abroad dealing with any nursing question from an original point of view, according to length. The minimum payment is 5s. Contributions on topical subjects are specially welcome. Notices of appointments, letters, entertainments, presentations, and deaths are not paid for, but we are always glad to receive them. All rejected manuscripts are returned in due course, and all payments for manuscripts used are made as early as possible after the beginning of each quarter.**

### Nursing in Small Isolation Hospitals.

BY A SPECIAL CORRESPONDENT.

Considered merely as hospital administration pure and simple, the nursing in small isolation hospitals reminds one of nothing so much as the Caucus Race in "Alice in Wonderland," in which celebrated event each competitor started when he was ready, and finished when he thought fit.

### Public Indifference about Isolation Hospitals.

The general public take absolutely no interest in isolation hospitals, apparently regarding them as obligatory additions to the ratepayers' burden; and never troubling to find out for what purpose the money is required and how it is spent when obtained. It is curious to notice how every little detail of the administration of the large charitable hospitals is discussed in the daily papers, and every so-called scandal made the most of. No doubt it has a wholesome effect. Public opinion, when thoroughly roused, is generally healthy, and it is quite time that public opinion was roused on the subject of isolation hospitals. When the British workman is compelled by law (practically) to send his child to a fever hospital, the law should be compelled to provide that child with proper care and attention. Each Joint Hospital Board is a law unto itself. Therefore it sometimes happens that the entire staff is represented by a caretaker and his wife, the latter doing the "nursing." Or perhaps there may be one nurse in addition to a married caretaker.

### The Number of Nurses Needed.

Sometimes, especially in the case of a new hospital, a start is made with a trained matron and a suitable number of nurses for the care of enteric fever, scarlatina, and diphtheria patients, together with wardmaids and other necessary servants. For some time all the staff may be kept fully occupied, but by and by there may be only a few convalescent "scarlet" patients, and no enteric or diphtheria except for an occasional solitary admission. Then the Hospital Board discover that there are only, say, three patients and about twelve nurses and other persons to attend on them. They instantly propose to do away with the whole staff, and try the "caretaker" plan. It is no use trying to explain that an enteric or diphtheria patient may be admitted to-morrow, that you cannot mix up patients suffering from different infectious diseases. Also, that, although one nurse may be able to manage half-a-dozen "scarlet" patients, every case of enteric fever requires a day and a night nurse, and the same rule applies to diphtheria in the acute stage. The local doctors know the kind of hospital they have to deal with; that it is no use sending up cases of serious illness to be nursed by the caretaker, or even by the one nurse, and so the hospital is not used as much as it should be in the interests of the public health. On the other hand, there is no reason why public money should be wilfully wasted; and no good nurse cares to stay in a post if she feels she cannot earn her salary, which possibly explains the difficulty of getting reliable nurses in small fever hospitals. Matrons and nurses of inferior training and character are frequently employed, with disastrous results.

### The Scheme of Exchanging Nurses.

The proposed scheme of exchanging nurses will never work satisfactorily until the employment of probationers is discontinued. The bulk of the work is done by raw probationers at salaries of from £10 to £12 per annum. Small fever hospitals have no business with probationers; and if the exchanging scheme is started all the borrowing hospitals will want to borrow trained nurses, and all the lending hospitals to lend raw probationers. If the control of all the fever hospitals in one county could be transferred to a central body, and some idea of the probable number of nurses required could be obtained from a careful study of the number of patients in each hospital during the past five years, a staff might be got together, each nurse to have a fixed minimum of training, both general and fever, the latter to be obtained in a large hospital. The salary of the nurses would be an expense, but the nursing would be an improvement on the present system; the nurses to be drafted to each hospital as required, each hospital to have its own fully-trained matron. No central home would be required, as the nurses might remain in the hospital in which they were serving until needed elsewhere.

### Specialising Hospitals.

It has been suggested that the plan of specialising hospitals might do away with many difficulties. A county mapped out into districts of a certain area, with four hospitals to a district, one each for the treatment of small-pox, scarlatina, enteric fever, and diphtheria. The great objection to this scheme is, of course, the risk run by the patients having to be conveyed long distances. But many hospitals at present have to bring in patients a distance of ten miles in wretched, draughty, springless horse-ambulances without serious result. If a system of motor ambulances could be established, the patients could be removed longer distances. If this plan were adopted it might lead to the abolition of a crying evil—namely, the mixing of acute and convalescent patients, especially in the scarlatina wards. In a small hospital the provision of separate wards for convalescent patients is out of the question. In a large hospital devoted to patients suffering from the same ailment it would be quite feasible. Yet again, fever patients may be classified as medical cases, and medical cases suffer from being nursed in wards, the temperature of which on a winter's day can only be raised to 56° by the expedient of closing all windows and ventilators. A proposal to instal hot pipes to supplement open fires would not be entertained by the Hospital Board.

### Incidents in Isolation Hospitals.

The following incidents in isolation hospitals may serve to illustrate the results of the present style of administration. In one hospital there were acute cases of enteric, diphtheria, and scarlatina in separate wards, with separate day nurses. One night nurse was considered sufficient; she was told by the matron that the fresh air would disinfect her as she passed the short distances between the blocks.

A tramp, supposed to be suffering from small-pox, was sent to a small-pox hospital in charge of a married caretaker; at the end of a fortnight the medical man in attendance decided that the disease had been wrongly diagnosed, and that it was really one of enteric fever. As it was the only case in the hospital, he arranged to transfer it to the enteric ward of another institution. The nurse who arrived to remove the patient was met by the caretaker's wife with the remark, "Poor thing, he's been that bad the doctor said I wasn't to wash him nor make his bed, and I haven't, not all the time he's been here." The state of that patient can be better imagined than described.

In another hospital the enteric and diphtheria ward night nurses were in the habit of leaving their patients and walking across to the "scarlet" night nurse for a comfortable cup of tea in her block.

### Small-pox Hospital Nurses.

Small-pox hospitals are especially bad. One case is on record of the nurses having gone down to the village post-office every morning for their letters, with a cloak thrown over the dresses they wore in the wards. And in another instance the nurses wandered in and out of the village at their own sweet will, and one of them even went so far as to set up a flirtation with one of the farm hands.

Once sixty diphtheria cases were nursed by two charwomen, assisted by a probationer of a month's standing. The medical officer could not obtain the services of trained nurses, as the Joint Hospital Board would not pay for them.

Again, picture the horror of bringing an enteric patient five miles in an ambulance on a winter's night, the ambulance so dilapidated that no light was possible inside as the wind blew it out at once, and the rain came in through various crevices to such an extent that the patient had to have a mackintosh on top of his blankets to prevent them from being soaked through. The nurse, being accustomed to it, put up quite cheerfully with her dripping dress.

### Minor Nursing Amenities.

### A MATRON AS PATIENT.

Very many excellent nurses who never fail in great emergencies, and can be relied upon absolutely to carry out instructions accurately, are wanting in the small amenities of nursing—those little acts of courtesy, of thoughtfulness, which make so much difference to the happiness of their patients. To state the case simply, they fail to put themselves even momentarily in the place of the patient, and consequently never see things from his point of view. Now we should remember that the whole system of nursing exists primarily for the benefit of the sick. Efficient ward management and automatic regularity of routine may be highly desirable, but there is a danger of its being carried too far and the true interest of the patient lost sight of in the anxiety of the nurse to do her mechanical work as hurriedly as possible and to earn for herself the reputation of being a smart nurse.

Having recently had some experience as a patient in a large London hospital, I have been led to consider seriously whether some important points are not left out in our present system of training nurses. It is quite a different matter when one considers nursing from a patient's point of view. The relative proportions of things are altered; it is like looking through a telescope the reverse way. The trifling details of her work, over which the nurse is so particular, do not appeal to the patient, who, perhaps, has asked in vain for some trifling attention which the nurse is too busy to render. Nurses are too apt to overlook the necessity for rest of mind as well as body for their patients. The rush and hurry of ward work, the necessary study for examinations, the natural desire to acquire the technicalities, so to speak, of their profession, loom so largely on their mental horizon that the minor acts of courtesy and thoughtful consideration are apt to be blotted out and vanish in a mist of forgetfulness.

One hears a great deal in these days of the influence of the mind upon the body, and there is much truth in the theory. Nurses should cultivate the habit of showing a kindly interest in the well-being of every patient who comes under their care. The very expression of the nurse's face has a distinct effect upon the patient, whose illness has made him morbidly sensitive to every influence around him. They should remember that the environment of the sick person is narrow: the ward has become his little world; the nurse represents to him all he has dreamed of in his ideal of womanly tenderness. Let it be hers to live up to this ideal and to make the ward in some sense a home to every patient who enters it. Very few nurses really mean to be unkind, but they are in too great a hurry to stop to make the patient's pillows comfortable, and they depart like a flash of lightning from the ward, and the patient is left to take his chance of melting the heart of a nurse later on in the day. One so often hears the remark, "Really, I have no time to stop now," that it seems as if a patient's comfort were, after all, a very secondary consideration in the rush of hospital work.

I have heard a patient moan, "Oh, my back! Oh, my back!" long after the nurses had hurriedly and unskilfully made her bed and left her in greater pain than they found her. "Pity 't is 't is true." It were better surely to take a little longer over the work, and perhaps not get through so much, than to actually cause suffering to the unfortunate beings who are temporarily at our mercy. There are nurses who never remember to shut the door, and the helpless patient is left in a draught, which may have serious consequences; others bang it as though it were the door of a railway train and nerves had not been invented. I have myself been awakened out of my sleep by a thoughtless night-nurse who—as I was a new patient—desired to make my acquaintance! I wondered sometimes whether it would not have been better to leave the ward as it was than with brush and duster to disturb the poor patient who had fallen asleep after dinner, having slept very little during the night; also whether it was not possible for the night nurse, by the exercise of a little ingenuity, to invent some plan for opening the door noiselessly, so as not to waken a sensitive patient who heard every sound?

Then, too, it seemed a pity to have store-cupboards in the wards, so that incessant journeys were made during the day, and occasionally during the night, in search of packets of wool and other dressings. This involves a perpetual distraction, and the patient often longs in vain for just an hour or two of absolute stillness. These minor details of consideration are frequently overlooked altogether, simply from want of thought and because patients have a settled repugnance to making complaints. The nurse should find time to put the flowers in water that are given to her patient, and make an effort to remember that it is nice to have your tea brought to you steaming hot, especially when you feel weak and low. She should also notice whether the blind is flapping about in the wind or if the sun is shining strongly in her patient's face. Although he may be too weak to make known his wishes, he will be very grateful for small atentions such as the foregoing.

If nurses only heard the chorus of appreciation which is raised in praise of the kindly nurse when the doors close behind them, they would be more anxious to win gratitude from their patients. "Oh, matron, I am so sorry that nurse is gone, she had such a feelin' 'art," one poor woman was heard to say, in a burst of confidence, when the matron made her daily visit to the wards. I have personally the pleasantest recollections of the nurse who remembered my weakness for a special cup and saucer, and who always brought me a hot cup of tea. She never was too busy to attend to her patients, and, accordingly, was a general favourite among them.

One small matter deserves mention as it is so frequently neglected; I mean the manner in which food is administered. A feeder full of milk is usually placed in such a position that the patient cannot possibly reach it and feed himself without upsetting some of the contents over his person or the bedclothes. Any object which is placed higher than the patient's head should never be filled to the brim. With a little thought this discomfort might easily be avoided. It is like being "behind the scenes" for a while to be a patient one-

MINOR NURSING AMENITIES—*continued.*

self, and to realise how the bearing of a nurse may affect her patients. One wishes sometimes that marks were given for thoughtfulness and gentle, sympathising ways, as well as for brilliant displays of head knowledge or even skill in bandaging. Presumably the private nurse gets her ideas of what is proper and fitting in the demeanour of a nurse during the time she is undergoing training. It is, then, really a matter of importance that she should be taught the gentle art of moving about noiselessly, the necessity of keeping as far as possible her patient's mind at rest by neglecting nothing which will help his recovery, remembering that nursing is the handmaid of medicine, and that very often a nurse does more for her patient than the doctor can possibly do.

There is no more satisfying career for a womanly woman than attendance upon the sick, and if nurses will only remember that it is quite as important to be sympathetic as it is to be clever, they will raise to still higher honour the profession which Florence Nightingale did so much to ennoble, and which affords scope for the exercise of the finest qualities of heart and brain possessed by any woman, whatever be her social position. It should not be possible to say of any nurse that her work has had a hardening effect upon her. Happily, some of us know that it is possible to keep a tender heart to the end of our nursing days, or it would have been better for us never to have entered at all upon the work of nursing.

### Practical Hints.

We welcome notes on practical points from nurses.

### HOW WE FEED A SMALL FAMILY ON 8½d. PER HEAD PER DAY.

We are only a small household, consisting of myself, probationer, maid, and the district nurse, and, as a rule, four patients on full ordinary diet, never less than eight persons, sometimes one or two more.

Our average daily expenditure per head for nourishment is 8½d. The dietary is as follows :—

Breakfast (staff) : tea, bread and butter, fried bacon, marmalade; three times in a fortnight the bacon is replaced by eggs once, kippers, dried haddock once. Occasionally mushrooms, tomatoes, potatoes are served with the bacon. Breakfast (patients) : Tea, bread and butter, bacon once, eggs once, fish once during week. Dinner (entire household) Saturday : either sirloin of beef or leg of mutton roasted, potatoes, and another vegetable in season, milk pudding.

Sunday : cold joint, potatoes, salad (pickles for staff), fruit tart, and milk pudding.

Monday : remains of cold joint made into a stew with plenty of vegetables.

Recipe for stew : place a frying pan over the fire and put in pan two ounces of good beef dripping, when hot slice into it a moderate sized onion and fry a golden brown, then slice into pan any cold vegetables, an apple, if in season a few gooseberries, fry these a few minutes, then take a penny packet of Edwards' desiccated soup, dissolve contents in a pint of cold water, and add this to the vegetables in the pan and bring all to the boil. Then slice very thinly any cold meat, sprinkle with pepper and salt, and dust very thickly with flour, add to other ingredients and boil up once. Then take a good sized porridge pot or double saucepan and empty contents of frying pan into inner one filling the outer pan with water which should be kept just boiling for not less than three hours. This is a delicious compound and is much appreciated if properly prepared and an intelligent cook will soon learn to vary it by leaving out or adding different flavourings; thus one week if procurable a couple of handfuls of fresh green peas could be added, another week they could be omitted and a couple of sliced carrots take their place, and so on.

Bread and butter pudding :—

Tuesday : roast loin of mutton (or fillet), two vegetables, milk pudding.

Wednesday : boiled neck of mutton (or shank of leg) with carrots, turnips (or parsnips), rice, and suet dumplings (only sufficient water is added to prevent burning).

Junket and stewed fruit or custard and stewed fruit :—

Thursday : beef olives, two vegetables, milk pudding.

Friday : fish, potatoes, and milk pudding with fruit or jam.

This is the menu for a week, of course it is varied occasionally, sometimes on Sunday we have a chicken (as well as the cold meat), and we vary the joints sometimes, but only the very best English meat is used as well as only fresh butter and new-laid eggs. A couple of rabbits sometimes replaces the fish on Friday.

Tea (staff) : tea, bread and butter, jam, and cake.

Tea (patients) : tea, bread and butter (toast occasionally in winter), jam on alternate days.

Supper and lunch : patients have their choice of milk or milk and bread : cocoa and bread and butter, soup, cornflour, arrowroot or Benger's food, etc., etc.

Supper for staff consists of cold meat and fried potatoes twice, rice and curry once, tinned tongue or pressed beef once, eggs once, fish once, stew as above once, jam and biscuits and tea in addition. Cocoa or coffee if preferred.

The patients on two-hourly feeds have, if ordered it, milk only. If allowed they have in addition to milk, beef-tea, plasmon, junket, Benger's food, cornflour, milk jelly, egg beaten up with milk or hot water. I try to give as varied a diet as possible. The most expensive article I consider to be beef-tea.

As ours is such a small institution we have no contracts but pay the full ordinary price and we have to buy from each tradesman in the town in rotation; that is to say, it is impossible to buy in the cheapest market. I find it the truest economy to buy only the best of everything and as far as possible get just the quantity I am likely to require when the articles are perishable. I arrange that there shall be absolutely no waste and try to manage that, however plain the fare may be, the cooking shall be as near perfection as possible.

### Wedding Bells.

A pretty ceremony was witnessed on Saturday afternoon, October 7, at the Baptist Church, Byron Hill, when Miss Florence M. Wilson, one of the district nurses of Harrow-on-the-Hill, was united in matrimony to Mr. Pitt-Howarjee, of Simla. The bride was given away by her mother, and the church was packed with patients and friends from the district, most of whom were afterwards entertained to a tea in the schoolroom. The event in the schoolroom was the presentation to the bride of a complete set of silver-backed brushes, hand-glass, scent-bottles, etc., subscribed for by old patients. She will be greatly missed in Roxeth and Harrow, where she has laboured for nearly seven years. Before coming to Harrow she was for some years a Queen Victoria Jubilee nurse at Freshford, near Bath, and was one of the favoured nurses who were received in audience at Windsor Castle by the late Queen. She will carry with her many good wishes from her old district to the new life which awaits her out in India.

### The New Home at the London Hospital.

PRESENTATION TO MISS LÜCKES.

Just behind the London Hospital, sheltered from the roar and bustle of the main road, stands the new Nurses' Home.

It is an imposing building, consisting of five stories, the interior being most artistically decorated and arranged.

On the ground floor there is a charming receiving-room for the nurses' friends, the walls being panelled half-way in dark oak, finished above in a pretty shade of red paper.

The sitting-room is large and most luxurious. At one end, through an alcove supported by two oak pillars, is the writing-room. The walls are distempered white, and also set in oak panels. There are a number of large crescent-shaped lattice windows in the room, and the three large fire-places are most quaint and beautifully designed. The floor is of polished boards, and the whole has the effect and atmosphere of a room in an ancient country house. At one side of the sitting-room, in a conspicuous place, hangs a carved oak board bearing this inscription in gold letters :

" By the unanimous vote of the House Committee it was resolved to associate the name of the matron with this Home. And thereby to commemorate the loyal and devoted service given by Miss Eva Lückes during 25 years to the London Hospital and to the improvement of nursing.

" This vote was passed in the hope that Miss Eva Lückes' name and work may be gratefully remembered, and in the belief that the high standard which she has established will ever remain as a tradition and example to the nursing staff of the London Hospital."

On each story in theHome there are thirty-four bedrooms, the colouring of the rooms being pale green, and pretty green linoleum covers the floor. The windows here also are of the lattice type, and make a charming effect.

All the bedrooms are furnished in dark oak, the wardrobes made to fit in cornerwise; an arrangement which economises space and adds prettiness to the whole, besides placing the glass door of the cupboard in a good light.

Besides mere necessities, each room contains a small oak writing-table fitted at one side with three drawers.

All the bedrooms are furnished alike, but those of the sisters are slightly larger, and contain, in addition, an arm-chair and a pretty little dark oak four o'clock tea-table; moreover they have large windows and a fireplace, as they are intended for bed-sitting rooms.

The corridors and stairs are heated with hot pipes. Although the Home is at present unoccupied and not fully furnished, it feels quite warm and airy. There are copious linen-rooms, boot-rooms, and four bathrooms on every floor, so the usual scrimmage for the morning tub will hardly be a dangerously heated contest. At one end of the Home there is an arrangement by which the nurses can always have boiling water for tea. There are lifts to be worked by the nurses themselves.

Electric light prevails, of course, and altogether an air of comfort and charm.

### The Presentation.

The presentation which the nursing staff, past and present, have made to Miss Lückes, in token of their esteem and love on the occasion of her completing 25 years' service at the Hospital, consists of a very handsome light-brown crocodile leather dressing-case, which bears Miss Lückes' initials, E.C.E.L., on the outside. The inside is lined with brown moiré silk, and fitted with thirteen cut glass bottles, brandy flask, soap jars, salve jars, etc., all of which are mounted in gold and bear Miss Lückes' monogram. It also contains a complete set of toilet requisites in tortoise shell, also having the monograms engraved; a comb-case, blotter, instrument-case, card-case, pin-box, gold corkscrew, address-book, jewel-case, and small work-case containing a gold thimble. The cases are made of brown seal leather, and some of these items were made specially. In one pocket a little gold travelling clock reposes.

To protect the crocodile case there is an outer case of canvas strapped with leather and lined with velvet. Nothing either by way of beauty or usefulness seems to have been forgotten, and the case is indeed a substantial and fitting proof of the esteem and admiration in which Miss Lückes is held.

### Miss Amy Hughes at the Church Congress.

The question of district nursing in town and country was introduced at the Church Congress last Friday night by Miss Amy Hughes, General Superintendent of Queen Victoria's Jubilee Institute for Nurses.

Miss Hughes commenced her paper by observing that the work and aims of district nursing (nursing the sick poor in their own homes) were now so universally accepted that any explanation or justification of its principles was unnecessary. She preferred to describe their practical application. The majority of associations already established had accepted conditions of affiliation with the Queen's Institute, which was now the largest existing organisation of district nursing, and had accumulated a wide experience of the work in all parts of the country and under all conditions. This experience had led to the establishment of two fundamental principles—special training of its nurses and expert supervision of their work. It was the express wish of her late Majesty that only hospital trained nurses should be entered on the roll of Queen's nurses, " in order that skilled nursing might be within reach of the poorest of her people." In addition to full hospital training, the nurses were taught under a superintendent of a nursing home to adapt their nursing knowledge to the circumstances of their patients, and to make the best of the unfavourable conditions and limited appliances available in a poor home. Many Queen's nurses were midwives, especially in country districts, and all were instructed in monthly nursing. There was also a system of inspection by expert Queen's nurses. This was often looked upon at first with suspicion as savouring of interference; but such fears soon proved to be groundless. There was no interference with local effort, no curtailing of personal energy, but simply a regular visitation of every affiliated association, from the city, with its forty or fifty nurses and their superintendents in various homes, to the solitary nurse in the remoteness of the country. Each association kept its reports, etc., on the same lines, and from each the same standard of work was required. The inspector came as a friend alike to the nurse and the committee, visiting the cases, seeing the books, and helping by her experience and advice to smooth over any little difficulties that might arise. It was the evenness of the work thus obtained that was making it a success by securing a uniform standard. The rules that the nurses shall not be almoners, and shall not interfere with the religious views of their patients or their friends lifted the work of Queen's nurses above suspicion of almsgiving and proselytising. At the same time the nurses were left free to bring their patients in touch with the local agencies that made for good.

Miss Hughes dealt at some length upon the methods of raising funds for the work, and proceeded to say that in many mining, colliery, and manufacturing centres, where there was a uniform level of work and wages, it was possible to support an association on the lines adopted by some of the provincial hospitals, *i.e.*, the men employed in the various industries agreed to the deduction of a certain sum—½d., 1d., or 1½d. weekly, fortnightly, or monthly—from their wages. Such a system with representatives of the various trades on the committee worked well, and there was more than one instance of an association which was thus almost self-supporting. If medical clubs existed in the district it was better that the payments to the nursing funds should not be arranged on exactly the same lines. It was not always possible to make such an association entirely self-supporting, and the funds might need supplementing by annual subscripitons and donations from the wealthier residents by church collections, and the other sources suggested for urban districts. It was better that an association should depend for its funds on the co-operation of all in the district rather than on one or two generous donors, as, should their contributions be unavoidably lessened or withdrawn, the association probably had to be given up, and those who had come to depend on the nurse's services suffered a great deprivation. In districts where midwifery was undertaken the fees charged for such cases were another small source of income to the association.

In conclusion, Miss Hughes averred that it was not systems alone, admirable as they might be, which brought success. It was the work of each individual nurse which made the work what it was. The influence of a good nurse remained after her nursing services were ended. It was the opportunities given by district nursing that made it so important and so responsible. Nurses who grasped the inner meaning of their work had few limits to their powers of usefulness. They nursed the homes as well as the patients, they gave valuable object lessons in the practical details of nursing, simple sick cookery, cleanliness, etc., thus helping their fellow-women to be less helpless and hopeless when sickness invades the home. They could advocate self-restraint, thrift, and household economies; they could give valuable advice in the dieting and management of infants and young children, so helping to strengthen the sinews of the nation. "As the child is, so the man is," and the simple truths of proper feeding taught in language "understanded by the people" meant the future welfare of its sons.

In the discussion which followed Mr. George Emery said that at a time when medicine was somewhat discredited, nothing was more important than the assistance of trained nursing. It was part of the nurse's duty to instruct the relatives in doing nursing work. Some of his remarks did not meet with approval, but when he urged that everybody should be taught something about nursing, as one never knew when an emergency might arise, the audience showed their appreciation by applause.

Captain Acland dealt with the mode of supplying nurses to the poor in Dorset. The system in vogue for several years where he lived was quite different in its working from that described by Miss Hughes in the Queen Victoria Jubilee Nurses' Institute. In small villages nurses were not always wanted, so that it was found more convenient and more economical to have a Central Home in the town, where a nurse could be sent for when required. With regard to fees, to the poor only 1s. a day was charged, but the rich paid the full fees, and they went to supplement the payment of the poor. These nurses were only meant for really serious cases and not for minor complaints. In his opinion poor persons were even more dependent than rich upon the well-trained nurse.

### Presentations.

Great Yarmouth General Hospital.—Miss E. R. Diver, on resigning the post of matron of Great Yarmouth General Hospital, which she has held for ten years, in order to accept the appointment of lady worker in the parish under the Vicar, has been presented with a purse containing money from the committee, a set of silver brushes and mirror from the hon. medical staff, a silver photograph from the house surgeon, and a silver rose bowl from the nursing staff, besides other tokens of regard from private friends.

### Nurses' Missionary Union.

On Wednesday last week there was an interesting meeting, held in connection with the Nurses' Missionary Union, on the occasion of bidding farewell to four of the members of the Union who are sailing this autumn to take up missionary work abroad.

Mrs. Druitt (*née* Radcliffe), who was trained at the Seamen's Hospital, Greenwich, is going to Hausaland, Northern Nigeria; Miss Everard, trained at Charing Cross Hospital, to Sierra Leone; Miss Thomas, trained at the Bristol General Hospital, to Fuh-Kien; and Miss Cropper, trained at the Leytonstone Infirmary, to Hankow.

By the kind invitation of Dr. and Mrs. Habershon, a large gathering of nurses and friends of the Union met in their house in Harley Street at 7 p.m. After "tea and coffee" the meeting began, and Mr. Pearce Gould, M.S., though having only just returned from a long journey, kindly presided.

The special interest of the occasion was centred in Dr. and Mrs. Arthur Druitt, who are sailing on Saturday for Hausaland, as Mrs. Druitt will be the first lady missionary to go into this comparatively new mission field. She has been a member of the Nurses' Missionary Union for nearly two years.

Dr. Druitt gave an admirable account of the people of Hausaland and their customs, and the medical missionary work which Dr. Walter Miller and he have been able to carry on among them, amid many difficulties and in spite of much opposition and prejudice. Then, the Secretary having said a few words about the work of the Nurses' Missionary Union and its motto, "The Evangelisation of the World in this Generation," Mrs. Druitt, Miss Cropper, and Miss Thomas each gave a message to their fellow-nurses, and spoke of the help and encouragement it had been to them to belong to the Nurses' Missionary Union, and of the joy they had in now being actually on their way to the foreign mission field.

Miss E. van Sommer, who has several times visited West Africa to cheer and help the missionaries in their varied work, then gave the farewell address, which was full of counsel and encouragement.

### Death in our Ranks.

We regret to hear of the death of Miss Elizabeth Godby, sister at Guy's Hospital, who passed away at her home in Dorking last Friday. She entered Guy's in 1887, and worked there after her training successively as sister of Luke and Charity wards, and for the last five years of Philip and Addison. She was thus well known to many generations of Guy's students and nurses. She was a pattern to everyone in her devotion to her work, and her upright character won her universal respect. Miss Godby returned from a month's holiday in August, but was evidently ill, and after a few weeks she went home, as it proved, to die. She was one of the secretaries of Guy's Missionary Union, an association which was very dear to her. It will be a long time before "Sister Addison" is forgotten by her old friends in the hospital.

The many friends of Miss Florence A. M. Van der Pant will deeply regret to hear of her death at Quetta, India, on September 8, from enteric fever. She was trained at the London Hospital, where she afterwards worked for some years on the private staff. She only went out to India last April, as a missionary nurse, in charge of the Church Missionary Hospital at Quetta, and the Society thus loses a faithful and devoted worker.

### The Central Midwives Board.

The first meeting after the summer holidays of the Central Midwives Board was held on Thursday last week. The members present were: the Chairman (Dr. Champneys), Mr. Ward Cousins, Dr. Dakin, Mrs. Latter, Miss R. Paget, Sir William Sinclair, Miss Wilson, and Mr. Parker Young.

The cases of three women desiring to be placed on the roll of midwives came up for consideration. The point at issue was: could they legally be said to have claimed admission before March 31? The first case was that of a woman who had applied for forms last year, and had then been unable to complete her training owing to an accident. This case was deferred. The other two cases referred to were women who had technically applied, not to the Board, but to the local supervising authorities, and through an unfortunate mistake and negligence their applications had not been forwarded to the Board before the expiration of the term of grace. The Board held that these women had applied for admission in due time and should therefore be enrolled, and suggested that these errors arose through the delegation of their authority by the county councils to the rural district councils.

A letter from the British Medical Association regarding the appointment of inspectors of midwives was merely acknowledged, the Board stating that it was beyond their province.

It was decided to continue to employ Messrs. Spottiswoode as printers of the minutes. Other tenders had been sent in, but were less satisfactory.

Dr. Dakin consented to be the medical member of the Board to assist the examiners in setting the papers for the ensuing examination.

It was decided that it was unnecessary to appoint visitors for the next examination, and that in future it would be sufficient to send a visitor to the centres from time to time only.

The question of the number and approximate dates of examinations to be held anually in London and the provinces, which had been fully discussed in July at a conference of the bodies concerned, was next considered. The Conference had reported that they thought that an examination in London every two months, and in the provinces every four months, would suit everybody. Sir William Sinclair held that the provinces and London should be treated alike. The Board felt that the question was too important to be decided at that meeting, and proceeded to settle the date of the next examination, postponing the further discussion of the matter until the November meeting. Tuesday, February 6, was the date fixed for the next examination. The Examination Committee, consisting of the chairman, Dr. Dakin, and Mr. Parker Young, was re-appointed.

### Belfast Maternity Hospital.

The most important business of the meeting was then taken. Mr. Ward Cousins (seconded by Sir William Sinclair) moved:—

> That the resolution of the Board of March 23rd, 1905, refusing the request of the Belfast Maternity Hospital for the recognition of its certificate as an approved qualification under Section 2 of the Midwives Act, be rescinded.

He said that in his opinion an act of injustice had been committed by the Board. The hospital was one of the best, and the women who wished to be enrolled on the strength of their certificate had had six months' training. The ground of the refusal was that the application came too late.

It appeared that the application for the recognition of the certificate was received on March 23, the day of the Board meeting, and that owing to pressure of time the business was postponed, and the hospital informed that their application was too late, though March 31 was the end of the term of grace. There were some fifty women who by the action of the Board were unable to be enrolled. There had been some correspondence with the Privy Council on the matter, and Dr. Byers, who represented the hospital, had written to the Council expressing willingness to come to an arrangement whereby those women who had claimed admission before March 31 should be enrolled. This, however, only accounted for about twenty of the number, and Mr. Ward Cousins argued that the Board were illegally treating the remaining thirty. The chairman protested that they were prepared to concede all that Dr. Byers, by his letter, requested. Both Mr. Cousins and Sir William Sinclair assured the Board that his letter was really only a minimum request, and that, from private letters and information, they were able to state that the hospital felt much aggrieved in the matter and would not in reality be satisfied if the Board merely admitted the twenty who had applied. Mr. Parker Young said that the other thirty women were in just the same position as many women who possessed the L.O.S. certificate and had not applied before March 31. On the contrary, Sir William pointed out that they stood in the position of the women whom, earlier in the meeting, they had admitted to the roll. There was an institution or body between them and the Board, and they ought not to be made to suffer. No doubt they were relying on the acceptance by the Board of the Belfast certificate. There was a good deal of heated discussion over the matter, and finally an amendment by Mr. Parker Young that the Board should act in accordance with Dr. Byers's letter was carried.

The report of the Standing Committee was then received and adopted.

Mr. Parker Young moved—

> That in future the minutes of all committees shall be kept and read at the next meeting, and put as a correct record of what passed at the previous meeting.

Miss Paget seconded, and the resolution was carried.

Miss Wilson moved—

> That the Secretary be instructed to communicate with the Local Supervising Authorities of England and Wales, asking them to furnish the Board with replies to the appropriate form of the accompanying lists of questions marked A and B respectively.

She said that she felt it was necessary that the Board should have as much information as possible about the way in which the work was being carried out in the country. In seconding, Miss Paget suggested that a question be inserted inquiring what arrangements were made for the payment of the medical practitioner if called in by a midwife, and that the councils be asked whether, in cases where there was no county medical officer, there was a medical adviser on the committee.

The resolution, with the suggestions incorporated, was carried.

### Everybody's Opinion.

[Correspondence on all subjects is invited, but we cannot in any way be responsible for the opinions expressed by our correspondents. No communication can be entertained if the name and address of the correspondent are not given as a guarantee of good faith, but not necessarily for publication. All correspondents should write on one side of the paper only.]

### THE PENSION FUND.

A Matron writes: I see a letter in your issue of September 30 *re* nurses receiving the money back which they had invested in the Royal National Pension Fund for Nurses. I thought that all lingering prejudices in the minds of nurses on that subject had been removed, but as the subject has been mentioned, I should like to state my experiences. I have been a member of the Fund for some years, and this year wished to withdraw my money, for private reasons. On one policy I had paid £47 7s. 4d. Within a month of my application I received a cheque for £53 12s. 2d. I also held two policies dating from the beginning of this year. According to the rules of the Fund, the money is not returnable until two years have elapsed from the date of issuing the policy. I received all my money back, less a small percentage for administration. I think these facts require no comment. I should like to add that all the time I have been a member the kindness and courtesy of Mr. Dick, the Secretary, have been unfailing. I wish all nurses realised how much he has their interests at heart.

### NURSES AND UNTIDINESS.

"Stella" writes: I agree with "M. J. L.'s" remark that "servants simply dread the advent of a nurse in the house"; but then this the nurse can easily overcome by her kindness and consideration for those around her. If she will make as little unnecessary work as possible, before she has been long in the house she would be looked upon as quite a comfort. Cleanliness, discipline, and tidiness are the groundwork of a nurse's training, whereas bandages, poultices, etc., are but minor details. As to the candle-grease upon the stairs, is it not a pity to carry these objectionable things about a house, where for a very small sum of money a useful little lamp can be purchased? To prevent the spilling of carbolic powder upon the lavatory carpet, may I suggest that it should be placed in a tall tin through which holes have been made, thus providing a dredger? As to the other breakables, they are often so well cracked before coming to the sick-room, that it is quite possible for a jug to leave the handle while it is in one's hand, for which we nurses take the blame most willingly. Finally, what nurse is not proud of a tidy room or ward when the doctor is expected?

### PLEASANT EVENINGS IN WINTER.

"A Superintendent" writes: It may interest some nurses in the country, especially those in remote districts, to know what some of their "co-workers" are doing in the town during off-duty time. Most district nurses are beginning to realise the necessity of a better acquaintance with maternity work, and even those who are certified midwives, though perhaps not working as such, often feel that there is much need to keep abreast of the times. It was early last winter that the nurses on this staff asked if I would give them some instruction in maternity work, taking, say, one evening during the week. I readily consented and began a regular course, taking, of course, the pelvis first. It occurred to me how much more professional, thorough, and altogether satisfactory it would be if the demonstrations were given by a medical man. I asked the doctor for the Home if he would undertake the course, and got a favourable answer, though we had to wait some weeks whilst he compiled a thorough and exhaustive series. He sent a pelvis, fœtal skull, and engravings for illustrations. Then I wanted a blackboard. I borrowed one from a school near, but as the doctor required it at each lecture it became inconvenient fetching and taking it back each time, so we had to think of something else. One of the nurses lent me a large drawing-board. I then got some black drawing-paper, and pinned the sheets one on each side of the board with drawing-pins. As each piece was used we pinned another on the top, so that all the diagrams were preserved. They were very valuable for future reference, particularly the fœtal circulation. We set the board up on a table against the wall, thus giving the doctor everything he required close at hand. The nurses were always in uniform for these lectures. They provided themselves with books and pencils for taking notes, and arranged themselves round the room. I must say that the picture always struck me as being very interesting. Anything not fully understood was noted down, and questions were written out and laid on the table at the beginning of each lecture. The questions were all dealt with in their proper course, and obscure points were thoroughly explained. A short time was allowed each evening for discussion, when the nurses could ask questions or speak of any particular case they had nursed. As the lectures went on it became necessary to have a "dummy" for positions. I made one myself of wool and black calico, which, though not perfect, answered the purpose very well. There were 14 lectures in all, and they were held once a week from 8 to 9 or 9.30 p.m. As a rule, the nurses also came to my sitting-room one evening in the week for further discussion, or to be questioned on the previous subjects. We were all quite sorry when the time came to an end; the lectures had roused considerable interest all round. Then came the question of acknowledgment—everybody felt most indebted. I happened to know that the doctor was passionately fond of chess, so concluded that a present would be very appropriate. The nurses all contributed, and we sent him a handsome set of "Staunton" chess men and board. Of course he was very much surprised, but much more delighted. Next winter I intend to have another set of lectures, but I have not yet decided on the subject.

### Appointments.

[No charge is made for announcements under this head, and we are always glad to receive and publish appointments. The information, to insure accuracy, should be sent from the nurses themselves, and we cannot undertake to correct official announcements which may happen to be inaccurate. It is essential that in all cases the school of training should be given.]

Ampthill Isolation Hospital.—Mrs. M. Gooch has been appointed nurse matron. She was trained at the Borough Hospital, Halifax, and Stockport Infirmary, and has since been nurse matron at Taunton Isolation Hospital.

Ashburton and Buckfastleigh Cottage Hospital.—Miss K. Marguerite Waydelin has been appointed matron. Miss M. Owen has been appointed assistant nurse. Miss Waydelin was trained at the Evelina Hospital for Children, Southwark, and the Warneford Hospital, Leamington. She has several times been matron's *locum tenens* at the Hinckley Cottage Hospital, and has done private nursing at home and abroad. Miss Owen was trained at the Tewkesbury Hospital and the Warneford Hospital, Leamington, where she has since been sister of the female medical and surgical wards. She has also done private nursing at home and abroad.

Birmingham General Hospital.—Miss Terry has been appointed night sister, and Miss McFarlane home sister. Miss Terry was trained at the Evelina and King's College Hospitals, London, and has since been ward sister at the Royal London Ophthalmic Hospital, and ward and housekeeping sister at the National Hospital for the Paralysed and Epileptic, Queen's Square, London. She also served for four years in South Africa as a member of the Army Nursing Service. She holds the certificate of the British Lying-in Hospital, London. Miss McFarlane was trained at the General Hospital, Birmingham, where she afterwards became theatre staff nurse. She has since been ward sister at Kidderminster Infirmary, and sister of male surgical ward and out-patient department at the General Hospital, Birmingham. Previous to her general training she was for two years nurse at one of the fever hospitals under the Metropolitan Asylums Board.

Brighton Hospital for Women (Portland Road Branch).—Miss E. F. Broodbank has been appointed assistant matron. She was trained at Camberwell Infirmary, where she was afterwards sister. She has since been engaged in private nursing. She has also had experience at the London Hospital, and was staff nurse at the Hospital of St. Cross, Rugby. Latterly she has taken divisional nurses' holiday duties at the Royal Hospital for Incurables, Putney Heath, and matron's holiday duties at Bute Hospital, Luton. She is registered under the Central Midwives Board.

Clapham Maternity Hospital.—Miss E. M. Cancelloi has been appointed sister. She was trained at the Royal Surrey County Hospital, Guildford. She is registered under the Central Midwives Board.

Deanham Workhouse, Holmfirth.—Miss Ellen E. Hill has been appointed charge nurse. She was trained at Stroud Union Infirmary, and has since been assistant nurse at Bromsgrove Union and charge nurse at Preston Union.

Great Northern Central Hospital, London.—Miss E. Hounsfield has been appointed sister. She was trained at tht Derbyshire Royal Infirmary and has since been sister at the Children's Hospital, Birkenhead, and the General Hospital, Northampton.

Grimsby and District Hospital.—Miss E. M. Lawe has been appointed sister of the Male Medical and Children's Wards and Out-Patient Department. She was trained at the Hartlepools Hospital, where she was also charge nurse, and has since been staff nurse at the Royal Victoria Hospital, Belfast. She has also done private nursing.

Infirmary, Whipps Cross Road, Leytonstone.—Miss Alma O. Anett has been appointed charge. She was trained at Chorlton Infirmary, West Didsbury, Manchester, and has since been sister in charge.

Llantrissant and Llantwit-Fardre Isolation Hospital.—Miss Mary J. Thomas has been appointed matron. She was trained at the General and Eye Hospital, Swansea, and she has since been charge nurse and holiday night superintendent at the Park Fever Hospital, London, holiday sister at the Plaistow Hospital, sister at the General Hospital, Merther Tydfil, and sister at the General and Eye Hospital, Swansea.

Royal Cornwall Infirmary, Truro.—Miss Phoebe Petrie has been appointed sister. She was trained at the Mildmay Mission Hospital, London, and the Royal Devon and Exeter Hospital. She has since been night charge nurse at "Friedenheim," Swiss Cottage, London, and charge nurse at the Miller Hospital, Greenwich.

Swallow Nest Hospital, Sheffield.—Miss M. J. Jones has been appointed charge nurse. She was trained at Kidderminster Infirmary and the Children's Hospital, and has since been nurse at the Western Hospital, Fulham, and staff nurse at the Allt-yr-yn Hospital, Newport, Monmouthshire.

Waterford County and City Infirmary.—Miss Mary R. M. McDowell has been appointed superintendent nurse. She was trained at the Sunderland General Infirmary, where she was afterwards charge sister. She has since been sister-in-charge of the Dublin Orthopædic Hospital, and superintendent sister of the East London Military Hospital, Transvaal, during the Boer War.

### Novelties for Nurses.

(By Our Shopping Correspondent.)

### EGERTON BURNETT'S DRESS MATERIALS.

Messrs. Egerton Burnett send a large selection of their autumn and winter patterns. The "Royal" serges, for which this firm is famous, not only fully retain their deserved good reputation, but seem, if possible, each year to improve with regard to colours, makes, weights, and moderate prices; and the variety and wonderful value offered this season enables me to pronounce them to be really ideal serges. A novelty for outdoor costumes is a strong material called "Wontarewilware," suitable also for boys' and men's wear, now introduced in a winter weight at 5s. 11d. per yard, 54 inches wide. There is a large assortment of tweeds, checked and striped cloths, and habit cloths, to choose from; and among them I must mention the Epsom, a good cloth, in various colourings, at 1s. 2½d. a yard, 48 inches wide. A tweed called "The Kew" is to be had, 44 inches wide, for the modest price of 1s. 8½d. a yard; and I would draw the notice of nurses to this inexpensive material, which would make charming coats and skirts. Messrs. Egerton Burnett make up their materials to order in their dressmaking and tailoring department. The firm supplies an unlimited variety of materials for nurses' dresses and cloaks, and washing dresses are to be had made to order complete from 15s. 11d. Nowhere are better, stronger, prettier, or less expensive nurses' dress materials to be found. There is also rather a unique selection of black dress materials of all kinds, while the motor cloths and tweeds for warm capes and coats, etc., are the very things to invest in at this season. A speciality of the firm is a pic-nic rug in art colours at 3s. 11d. This rug washes, and is useful for travelling and many other purposes. Always admirable, Messrs. Egerton Burnett's silk materials this year seem prettier and more uncommon than ever. Nurses wishing for pretty blouses or for evening dresses for off-duty wear would be charmed with them; also with the very pretty embroidered blouse flannels. Altogether I can thoroughly recommend any nurse to write for patterns and catalogues from this reliable firm, before deciding upon any new autumn or winter garments.

### THE CONDIMENTS AND OTHER PRODUCTS OF MESSRS. LAZENBY.

Lazenby's is one of those old-fashioned firms which have survived the invasion of the modern manufacturers by reason of the intrinsic excellence of their productions. They have not descended to the level of those firms which, in order to meet with the demand for cheap provisions, have made use of inferior materials. Harvey's Sauce is still made according to the old recipe, and is as excellent to the palate as ever. Medical opinion may be averse to the use of condiments, but the taste for them is so general that it is necessary to accept the fact that they will probably always find purchasers. It is wise, therefore, to recommend the use of the best only, and to advocate the advantage of purchasing from a firm with an established reputation. Messrs. Lazenby prepare all manner of comestibles. Their chef sauce and soups in tablet form are especially to be commended.

### AN ASEPTIC WALLET.

Nurse Barry has invented and patented an aseptic wallet to supersede those made in leather or other materials which do not wash. The aseptic wallet is neatly made of white piqué and bears a red cross in turkey twill. It is attachable to the waistband by a double flap, which is of the same form as adopted in leather wallets, and this flap is fastened by a button with shank to the waistband, so that it is secure yet easily detached. It is surprising that the old form of wallet has been so long countenanced by those who are the exponents of sanitation, and now that Nurse Barry has invented so admirable a substitute there is no excuse to use leather or other unwashable wallets. The antiseptic wallet can be procured from Messrs. Maw, price 2s.

### NURSES' REQUISITES.

Messrs. Brookes and Co., of 43 Borough High Street, have sent us their new catalog[illegible] for nurses. They have been induced to prepare this o[illegible]g to the many inquiries they have received from nurses si[illegible] they opened this special department. The catalogue co[illegible]ains illustrations and descriptions of cloaks, bonnets, dresses, and all the appurtenances connected with uniform and underclothing. The prices are extremely moderate.

### Notes and Queries.

**REGULATIONS.**

**The Editor is always willing to answer in this column, without fee, all reasonable questions, as soon as possible.**

**But the following rules must be carefully observed.**

**1. Every communication must be accompanied by the name and address of the writer.**

**2. The question must always bear upon nursing, directly or indirectly.**

**If an answer is required by letter a fee of half-a-crown must be enclosed with the note containing the inquiry.**

*South of France and Italy.*

(7) Will you kindly give me the addresses of nursing institutes in the South of France or Italy where I should be likely to get private work for six months?—*A. M. B.*

The Nice Nursing Institute, Villa Pilatte, Avenue Désambrois, Nice; English Nurses' Home, Villa St. Joseph, Biarritz; the San Remo Institute for Trained English Nurses, Sunny Bank, San Remo; the Anglo-American Nursing Home, 265 Via Nomentana, Rome; the Association of Trained Nurses and Masseuses, 7 Via Rondinelli, Florence. But it is too late now, we fear, for you to make arrangements for the present season.

*Diphtheria.*

(8) Will you kindly advise me of a good book on the nursing of diphtheria, especially the complications after anti-toxin, and the preparations for cases of tracheotomy?—*J. T.*

See Dr. Woolacott's lectures which have appeared in The Hospital, and which are shortly to be published in book form by the Scientific Press.

*Dispensing and Chemistry.*

(9) Would you kindly tell me where I could obtain full instruction in dispensing and chemistry in London?—*M. F.*

At Miss Buchanan's Laboratory, Gordon Hall, Gordon Square, London, W.C.

(10) Kindly tell me how and where I could learn dispensing, how long it would take, what the charge would be, and if I could learn it in my spare time?—*Probationer.*

See reply to *M. F.*, or write to the Pharmaceutical Society, 17 Bloomsbury Square, W.C.

(11) I am desirous of gaining a certificate or diploma for dispensing. Would you be so kind as to give me your advice?—*Anxious.*

(12) Can you give me all particulars about dispensing for a woman? What is the first step, the age to begin, and is it a remunerative profession?—*F. H. T.*

See replies above.

*Probationer.*

(13) Will you kindly give me any information as to where they would take me as a probationer in a large hospital after having had some months' training in a cottage hospital, and should I get any salary?—*C. R.*

In "How to Become a Nurse. The Nursing Profession: How and Where to Train" you will find full details of all you want to know.

*Monthly Nursing.*

(14) Will you please tell me if it is necessary for a trained nurse (not midwife) to have a certificate for monthly nursing? *N. E. W.*

Certainly, if she intends to nurse maternity cases.

*India.*

(15) Would you kindly give me some information as to the course I should take to go out to India to nurse? I am a trained nurse, and at present belong to one of the private nursing homes in London.—*Sister E. S.*

"How to become a Nurse. The Nursing Profession: How and Where to Train," published by the Scientific Press, Limited, Southampton Street, Strand, gives full particulars of military and civil nursing in India.

*Nutrient Enemata.*

(16) Will you kindly tell me whether albumin is digested by the bowel when given in a nutrient enema, as for example one consisting of egg and mily?—*K. R. B.*

Yes, a certain quantity of albumin is absorbed by the bowel from a nutrient enema.

**Handbooks for Nurses.**

Post Free.

"A Handbook for Nurses." (Dr. J. K. Watson.) ... 5s. 4d.
"Nurses' Pronouncing Dictionary of Medical Terms" 2s. 0d.
"Art of Massage." (Creighton Hale.) ... ... ... 6s. 0d.
"Surgical Bandaging and Dressings." (Johnson Smith) 2s. 0d.
"Hints on Tropical Fevers." (Sister Pollard.) ... 1s. 8d.
Of all booksellers or of The Scientific Press, Limited, 28 & 29 Southampton Street, Strand, London, W.C.

### For Reading to the Sick.

FOOD IN THE DESERT.

He led me through the wilderness,
A long and lonely way;
He soothed me with His tenderness,
And fed me day by day.
Oh, better far the wilderness
And desert way to me;
If wandering in its loneliness
I should be nearer Thee.

*The Dove on the Cross.*

Try to picture the scene before your eyes. Five thousand men, women, and children are in the desert following our Lord, eager to listen to His words and to see "the miracles which He did on them that were diseased," yet with no food or possibility of obtaining food. Our Lord is equal to the occasion. "Make the men sit down," He says, "and He took the five barley loaves," which a boy in the company happened to have, "and when He had given thanks He distributed to the multitude."

The meal is over, but our Lord sees fragments here and there, broken bits of bread lying about in disorder, that were "over and above" what was needed. But what is their value? Our Lord seems to set store by them, for He says: "gather up those fragments that remain, lest they be lost." It sounds so strange, yet a lesson of great encouragement is hidden in these words. "Lose nothing, despise nothing, however small, but gather up the fragments *lest they be lost*," for everything is of value to our Lord.

What can be picked up from my life? Failure seems stamped upon it, and I can do so little. Oh, say not so, for with God nothing is small; with God there is no such thing as failure; to God all is of value. With a father's eyes He looks at our lives, and when we say to Him with a blush of shame: "Master, I can offer Thee but a few fragments," the answer comes quickly: "Let them not be lost; gather up those fragments, they are dear to Me."

Our little deeds, our broken prayers, our small acts of conformity to God's will, our twinges of pain, our hours of desolation, these are fragments that accumulate; these go to make the furniture of our eternal home; they are of value, one and all, to the Sacred Heart of Him Who said: "Gather up the fragments that remain, lest they be lost."

*Anon.*

Be not anxious about little things, if thou wouldest learn to trust God with thine all. Act upon faith in little things: commit thy daily cares and anxieties to Him; and He will strengthen thy faith for any greater trials. Rather, give thy whole self into God's Hands, and so trust Him to take care of thee in all lesser things, as being His, for His own sake, Whose thou art.

*E. B. P.*

Then weep not o'er the hour of pain,
As those who lose their all;
Gather the fragments that remain,
They'll prove nor few nor small.
The thankful spirit finds relief
In calm submissive love;
Toils on in hope, amidst his grief,
And looks for joys above.

*Duncan.*

## Figures and Tables

**Fig. 1. f1:**
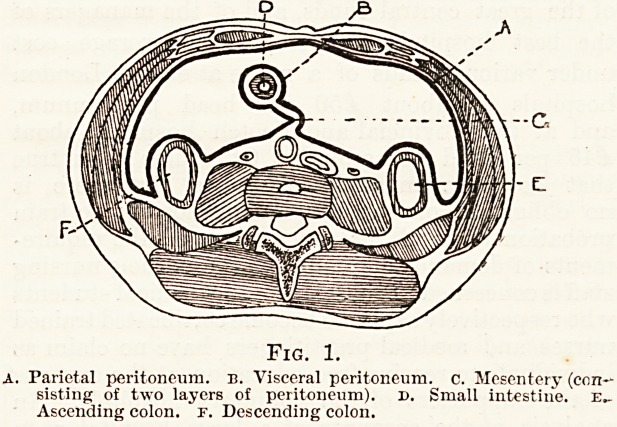


**Fig. 2. f2:**
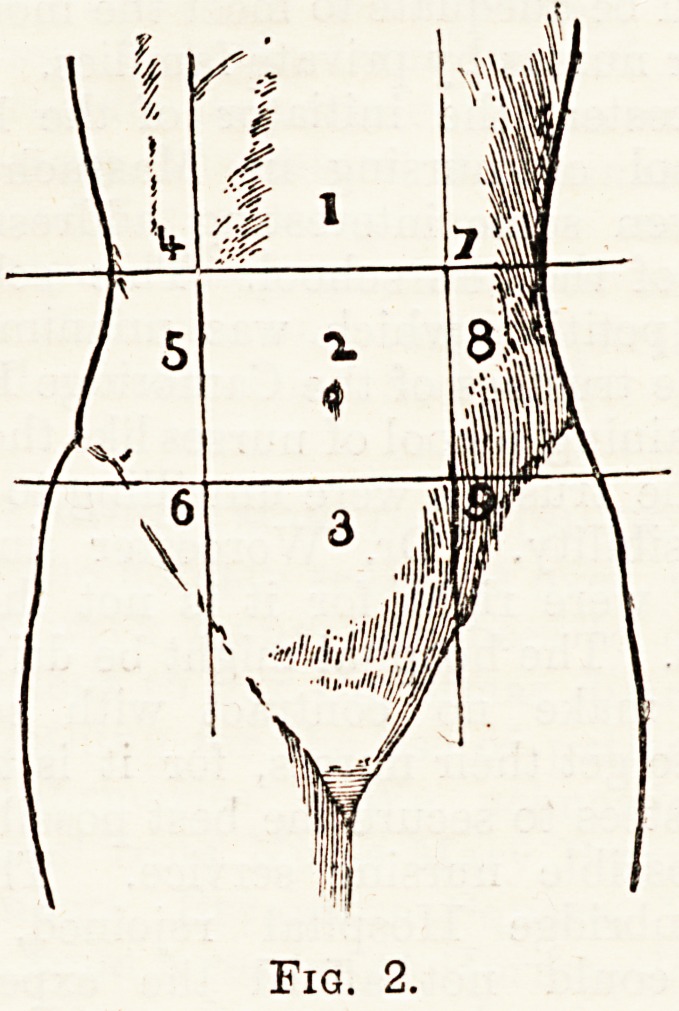


**Figure f3:**
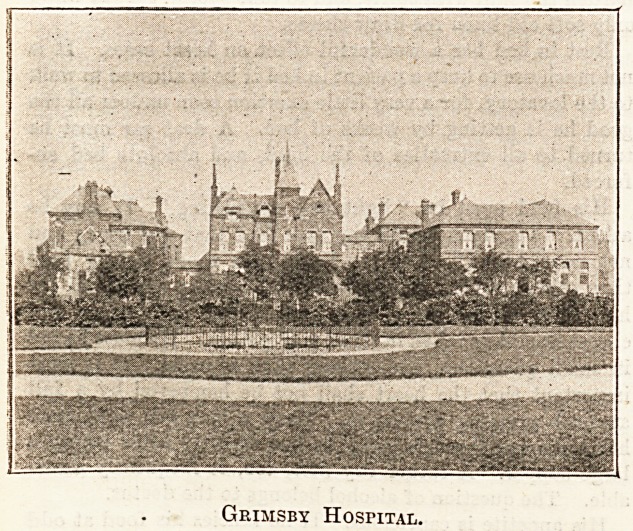


**Figure f4:**
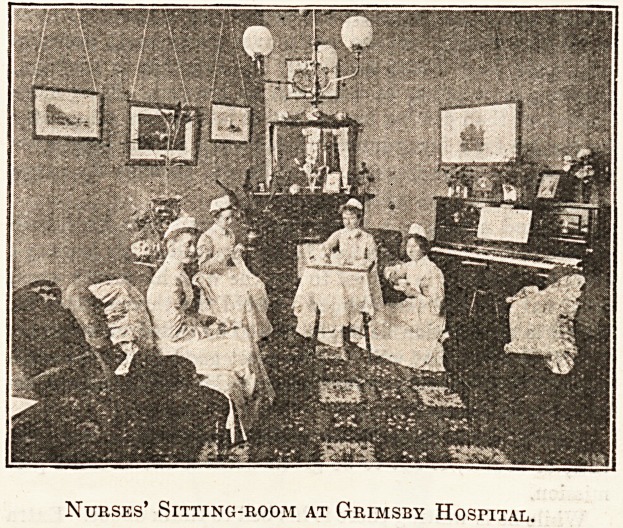


**Figure f5:**
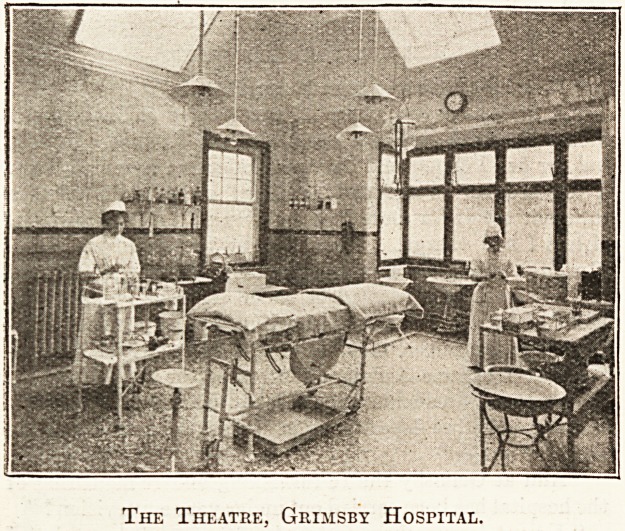


**Figure f6:**
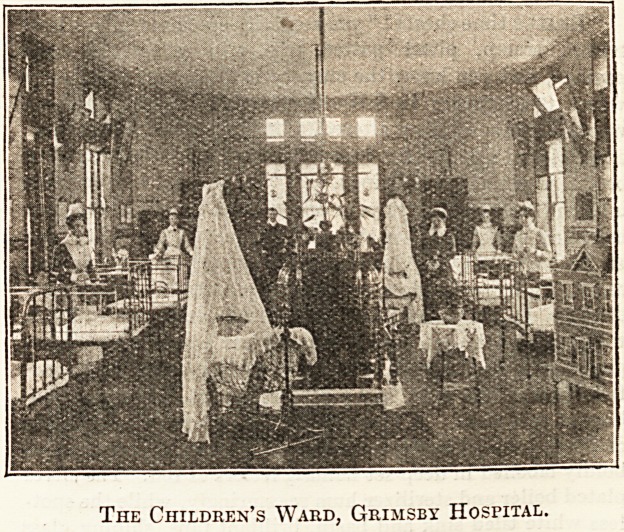


**Figure f7:**
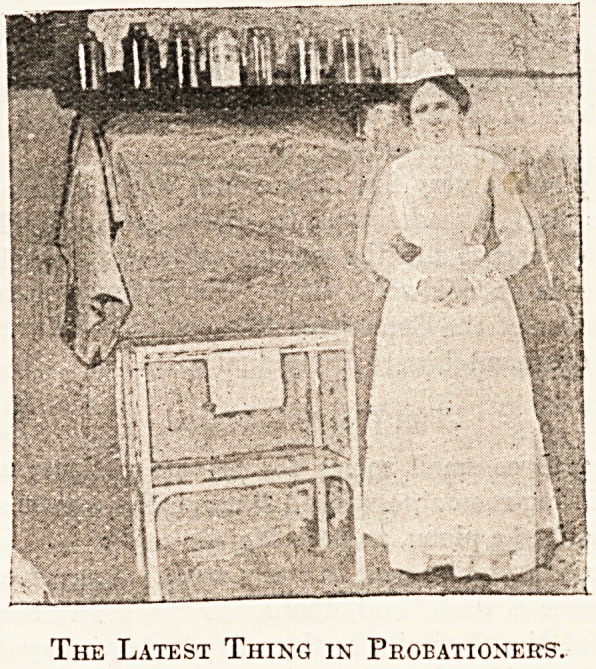


**Figure f8:**
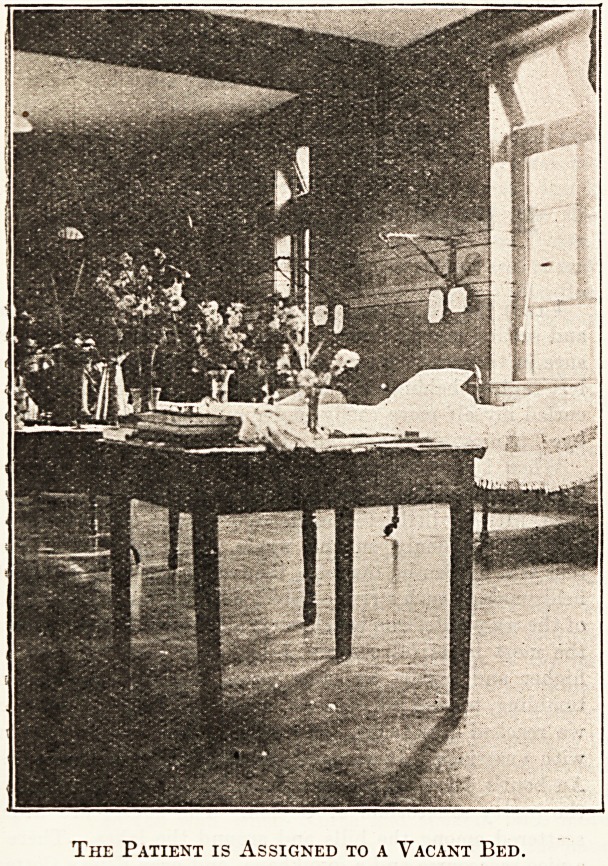


**Figure f9:**
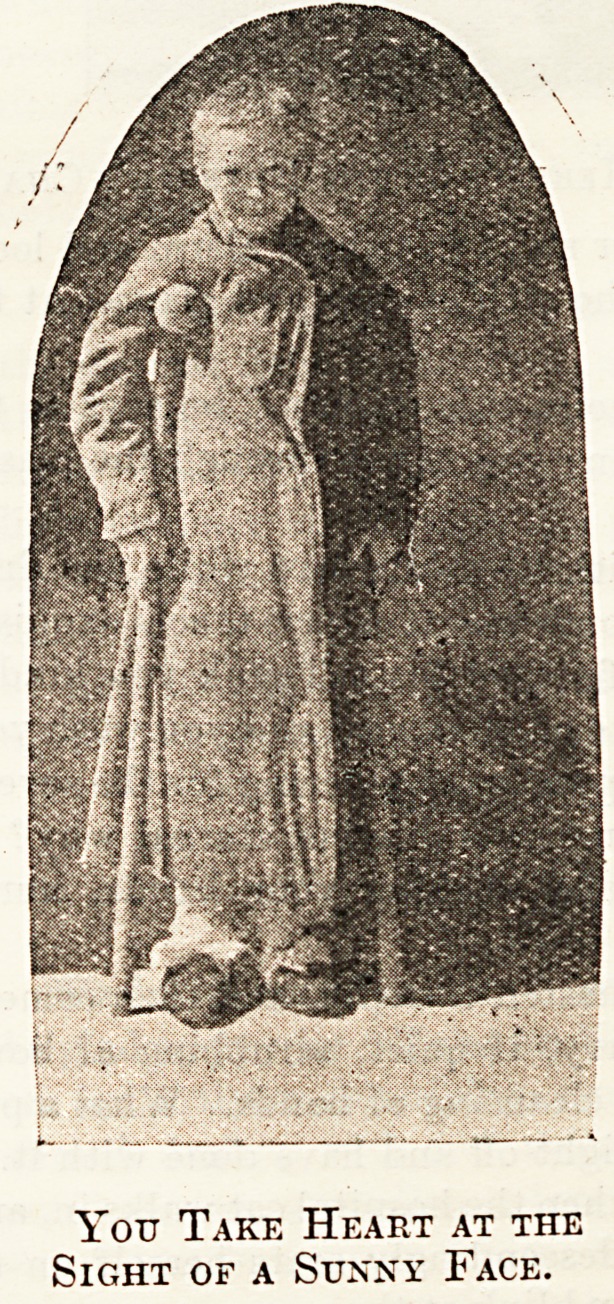


**Figure f10:**
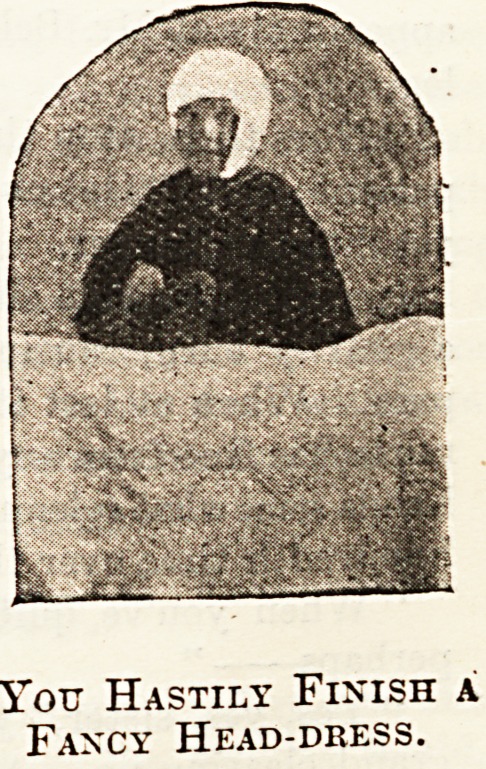


**Figure f11:**